# Evolution of the real area of contact during laboratory earthquakes

**DOI:** 10.1073/pnas.2410496122

**Published:** 2025-06-06

**Authors:** Baoning Wu, Sylvain Barbot

**Affiliations:** ^a^Department of Earth Sciences, University of Southern California, Los Angeles, CA 90089; ^b^Institute of Geophysics and Planetary Physics, Scripps Institution of Oceanography, University of California, San Diego, La Jolla, CA 92093

**Keywords:** earthquake, fracture mechanics, rupture dynamics, friction law, rate-and-state friction

## Abstract

Empirical slip-rate- and state-dependent friction laws and linear fracture mechanics constitute popular approaches to explaining earthquakes. However, the physics underlying friction laws remain elusive and fracture mechanics does not specify fault strength at the various conditions relevant to crustal faulting. Here, we introduce a physical constitutive framework that augments the traditional approaches by incorporating the real area of contact as the state variable. The physical model explains the dynamics of slow and fast ruptures on transparent materials, as well as the amount of light transmitted across the interface during laboratory ruptures. The constitutive framework elucidates the origin of empirical friction laws, and the simulated ruptures can be described by linear elastic fracture mechanics. Continuous measurements of the physical state variable or its proxies during seismic cycles emerge as a novel tool for probing natural faults and advancing our understanding of the earthquake phenomenon.

## Significance

The mechanisms controlling rupture dynamics during seismic cycles remain elusive. Empirical friction laws and fracture mechanics constitute useful frameworks for understanding dynamic ruptures. However, these approaches do not explain the evolution of the frictional interface. Here, we present a physical model of fault friction that simultaneously captures rupture mechanics and light transmissivity across the fault. We validate the model using laboratory measurements during velocity-step and dynamic rupture experiments in transparent materials. The physical model elucidates the origin of the slip-rate and state dependency of friction and predicts abrupt-weakening behaviors under particular parametric configurations, making it compatible with fracture mechanics analyses. Continuous monitoring of fault properties during seismic cycles may be key to improving our understanding of earthquakes.

Earthquakes can be interpreted as frictional stick–slip instabilities at the crustal scale (1, 2). Empirical rate-and-state friction laws have been developed in the past few decades to capture the phenomenon (3–5), and have become a prime framework for modeling the seismic cycle (6–8), capturing the various styles of ruptures and recurrence patterns of earthquakes (9–13). However, the phenomenological description of friction precludes extrapolation to the various kinematic, hydrothermal, and barometric conditions relevant to crustal faulting (14, 15). Meanwhile, linear elastic fracture mechanics explains the macroscopic characteristics of seismic ruptures in the laboratory and nature (16–21), providing a useful framework to understand earthquake triggering, nucleation, propagation (22–24).

In this study, we discuss how a constitutive model of fault friction simultaneously explains the evolution of frictional resistance and the physical state of the interface. We first introduce a constitutive framework that connects the empirical state variable with the real area of contact at the interface. We then analyze data from laboratory experiments on polymethyl methacrylate (PMMA), a transparent material that allows optical imaging of the real area of contact during all phases of the seismic cycle. We first calibrate the model with quasi-static velocity-step experiments (25). We then use fully dynamic seismic cycle simulations to explain the evolution of the real area of contact and optical transmissivity during spontaneous slow and fast dynamic ruptures (17).

While the simulations explain the rupture speed and stress drop of laboratory earthquakes, as also possible with the empirical friction laws and linear fracture mechanics approaches, the model reproduces the amount of light transmitted across the interface during laboratory ruptures. The numerical simulations produce an abrupt weakening profile near the rupture front, compatible with common underlying assumptions in fracture mechanics analyses. Furthermore, the relationship between rupture speed and fracture energy of the simulated ruptures adheres to the predictions of linear elastic fracture mechanics. Hence, the physical model reconciles and augments the traditional modeling approaches.

## Constitutive Friction Law

**Fig. 1.**
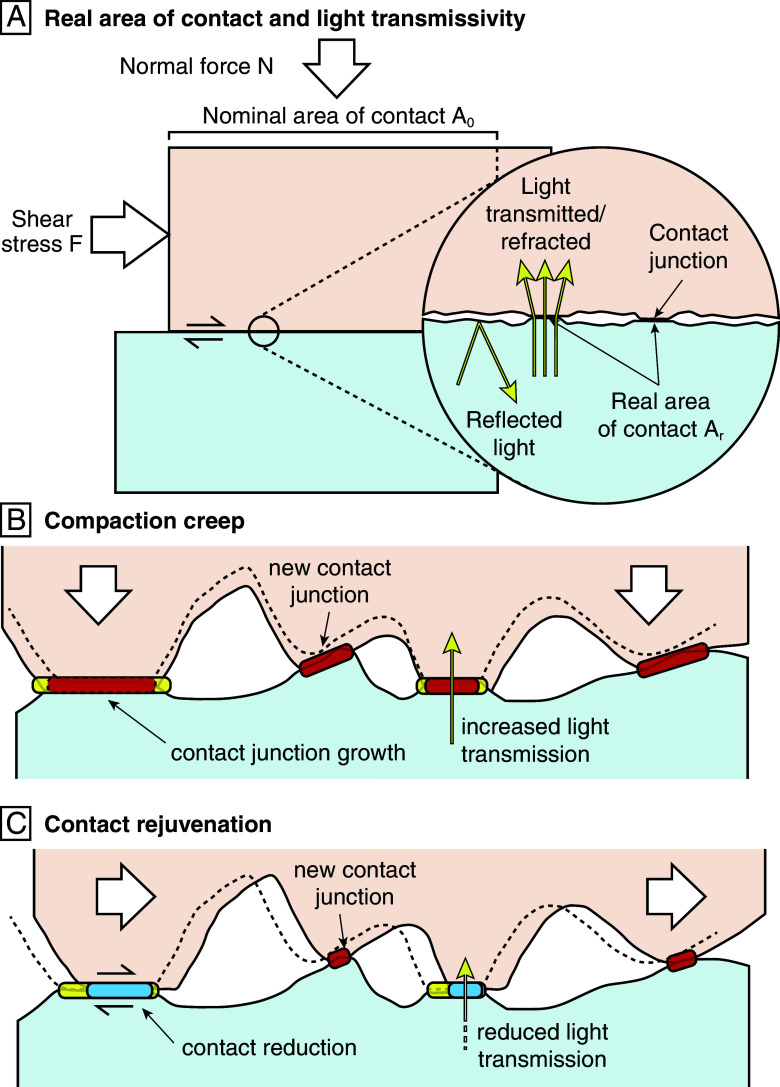
(*A*) Frictional sliding on a rough interface and modulation of transmitted light. Real contact occurs at small isolated junctions. (*B*) During compaction creep, at stationary contact or sufficiently low slip rates, the contacts flatten, transmitting more light across the interface. (*C*) Contact rejuvenation occurs during sliding, with the erosion of existing contacts and the formation of new ones. As a result, less light is transmitted across the interface.

We describe a physical constitutive friction law in isobaric, isothermal, and nominally dry conditions. Consider two solid, planar surfaces in bare contact with roughness at the microscale (Fig. 1*A*). A normal force N and a shear force F are exerted on the interface. The two walls enter into contact only at numerous small isolated junctions, and the real area of contact $A_{r}$ is significantly smaller than the nominal contact area $A_{0}$. Consequently, the actual normal stress at each contact is markedly greater than the macroscopic normal stress $\sigma =N/A_{0}$. To explain the empirical slip-rate dependency, we consider the shear rheology of the junction layer. Assuming that contact junctions experience a shear stress $\tau_{c}$ on average, the plastic strain rate follows (26)

[1] $$\dot{\epsilon }={\dot{\gamma }}_{0}(\frac{\tau_{c}}{\chi_{s}}{)}^{n}\;,$$

where ${\dot{\gamma }}_{0}$ is a reference strain rate and n≫1 is the power law exponent. As n is usually a large number under typical conditions (26), $\chi_{s}$ retains the physical meaning of a yield strength (27).

The local contact shear stress $\tau_{c}=F/A_{r}$ depends on the ratio between the net shear force and the real area of contact. Assuming that the junction layer thickness h does not change significantly, we can express the bulk slip-rate $V=2h\dot{\epsilon }$ as a function of the net shear force F as

[2] $$V=V_{0}(\frac{F}{\chi_{s}A_{r}}{)}^{n}\;,$$

where $V_{0}=2h{\dot{\gamma }}_{0}$ is a reference slip rate. To account for the empirical state dependency of friction laws, we consider the evolution of the real area of contact $A_{r}$. During compaction, contact junctions flatten with time due to inelastic deformation, and new junctions form (Fig. 1*B*). Macroscopic slip erodes existing junctions, and creates new ones, leading to contact rejuvenation (Fig. 1*C*). Therefore, the real area of contact $A_{r}$ depends on the contact lifespan θ (28). Assuming a power law, we describe $A_{r}$ as

[3] $$A_{r}=\frac{N}{\chi_{n}}(\frac{\theta }{\theta_{0}}{)}^{\frac{\alpha }{p}}\;,$$

where $N/\chi_{n}$ is the reference real area of contact when $\theta =\theta_{0}$, $\chi_{n}$ is the indentation hardness, α is the power-law exponent that describes the $A_{r}$ dependency on roughness, p is a power-law exponent that describes the inelastic rheology at the contact asperity, with α/p≪1 (*SI Appendix*, *Text* 4).

The above assumptions suffice for defining the slip-rate and state dependence of the friction coefficient μ=F/N. Combining Eqs. **2** and **3**, we obtain the constitutive friction law

[4] $$\mu =\mu_{0}(\frac{V}{V_{0}}{)}^{\frac{1}{n}}(\frac{\theta }{\theta_{0}}{)}^{\frac{\alpha }{p}}\;,$$

where $\mu_{0}=\chi_{s}/\chi_{n}$ is a reference friction coefficient, in accordance to adhesion theory (29, 30). Since 1/n and α/p are both positive numbers significantly smaller than unity, we can approximate Eq. **4** by its truncated Taylor series expansion as

[5] $$\mu \approx \mu_{0}+a\ln\frac{V}{V_{0}}+b\ln\frac{\theta }{\theta_{0}}\;,$$

where $a\approx \mu_{0}/n$ and $b\approx \mu_{0}\alpha /p$, and where we recognize the empirical slip-rate- and state-dependent friction law (5). Additionally, the age of contact is associated with an evolution law (3, 5), which falls into the aging-law end-member

[6] $$\dot{\theta }=1-\frac{V\theta }{L}\;,$$

where L is the characteristic weakening distance defining the characteristic contact time $\theta_{0}=L/V_{0}$, or the slip-law end-member

[7] $$\dot{\theta }=-\frac{V\theta }{L}\ln\frac{V\theta }{L}\;.$$

Determining which evolution law is most suitable to capture fault mechanics is an active area of research (31–37), but out of the scope of the current study. The physical framework connects the empirical state variable to the real area of contact through Eq. **3**, providing a pathway to infer the state variable from laboratory observations. Modulation of the real area of contact affects electric conductivity (38), hydraulic permeability (39), seismic transmissivity (40), porosity (41), dilatancy (42), and optical reflectivity (25), among other elements, across the interface. In the following, we focus on optical constraints on the real area of contact, which offer a more direct proxy than other experimental methods.

## Quasi-Static Evolution of the Real Area of Contact

**Fig. 2.**
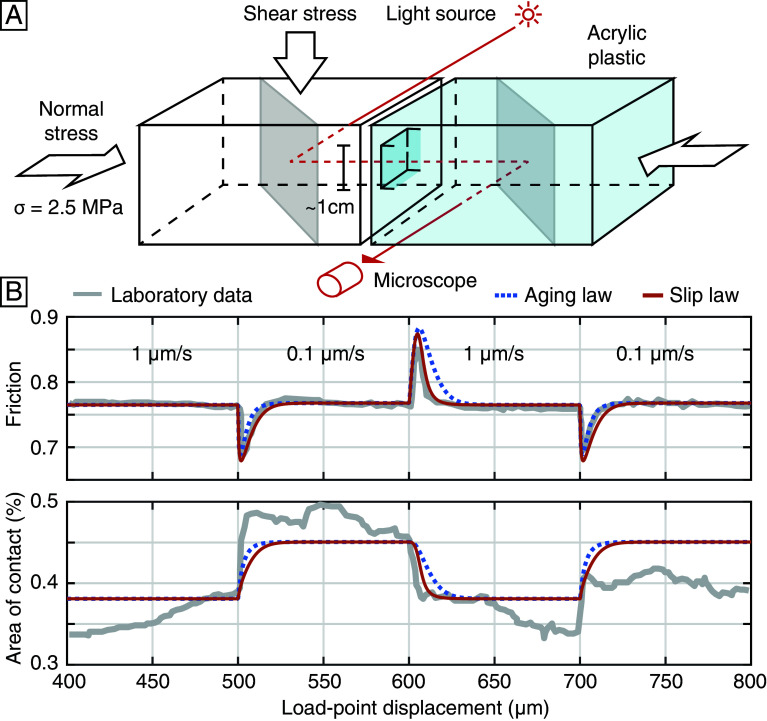
(*A*) Simplified schematic of the double direct shear apparatus with optical imaging of micro-asperities (25). The second steel loading block on the *Right* side is not shown. (*B*) Evolution of the frictional resistance and the real area of contact of PMMA during velocity steps (25) (gray line) and prediction from the constitutive model (dashed blue line for the aging law and orange line for the slip law).

The constitutive framework makes predictions that can be compared with simultaneous measurements of the frictional resistance and the real area of contact in a laboratory setting. We first consider double direct shear experiments on PMMA in quasi-static conditions, conducted by Dieterich and Kilgore (25) (Fig. 2). When incident light strikes the sliding interface, monochromatic light is transmitted only through the contact junctions. The intensity of the transmitted light can be utilized to infer the real area of contact $A_{r}$ during sliding. The slip rate is imposed on the sliding interface and is altered between 1 µm/s and 0.1 µm/s every 100 mm of slip. The resulting frictional resistance and the real area of contact density $A_{r}/A_{0}$ are measured continuously.

We simulate these data numerically considering a spring-slider assembly obeying the constitutive framework (*SI Appendix*, *Text* 1). Through trial and error, we first identify a set of constitutive parameters that best fits the laboratory friction data. The parameters $\mu_{0}=0.765$, a=0.0546, b=0.0558, L=6.80µm, and the spring stiffness k=0.10MPa/µm can be uniquely determined by matching the steady state friction at two different sliding velocities, the immediate friction response to the velocity step, and the friction evolution distance after the velocity step. The aging-law and slip-law end-members can reproduce the friction evolution to the first order (Fig. 2). However, previous studies suggest that the slip law better captures the symmetry of increasing and decreasing velocity steps (34, 35, 37).

We then predict the evolution of the real area of contact density using the relationship between $A_{r}$ and θ as well as the correspondence between the physical and empirical parameters

[8] $$\frac{A_{r}}{A_{0}}=\frac{\sigma }{\chi_{n}}(\frac{\theta }{\theta_{0}}{)}^{\frac{b}{\mu_{0}}}\;,$$

where $\sigma =N/A_{0}=2.5$ MPa is the macroscopic normal stress. The indentation hardness $\chi_{n}=659\;$MPa can be determined by fitting the baseline of the contact area. The difference with the instrumented value of 502±80MPa for PMMA (25), may stem in part from simplifying modeling assumptions as the frictional strength of PMMA is a nonlinear function of normal stress (43–46). The misfit to the real area of contact data may reflect the random distribution of asperities coming in and out of the imaged section during sliding. The model captures the transient evolution of the real area of contact during velocity steps and the resulting inverse relationship with slip-rate at steady-state.

## Evolution of the Real Area of Contact During Dynamic Ruptures

With increased instrumental capabilities over the past decades, confined elastodynamic ruptures have been imaged in a laboratory setting (47–51). Some experiments involving transparent materials have captured the spatiotemporal evolution of the real area of contact (17, 48, 52–54), offering a valuable opportunity to test the predictions of the constitutive framework.

**Fig. 3.**
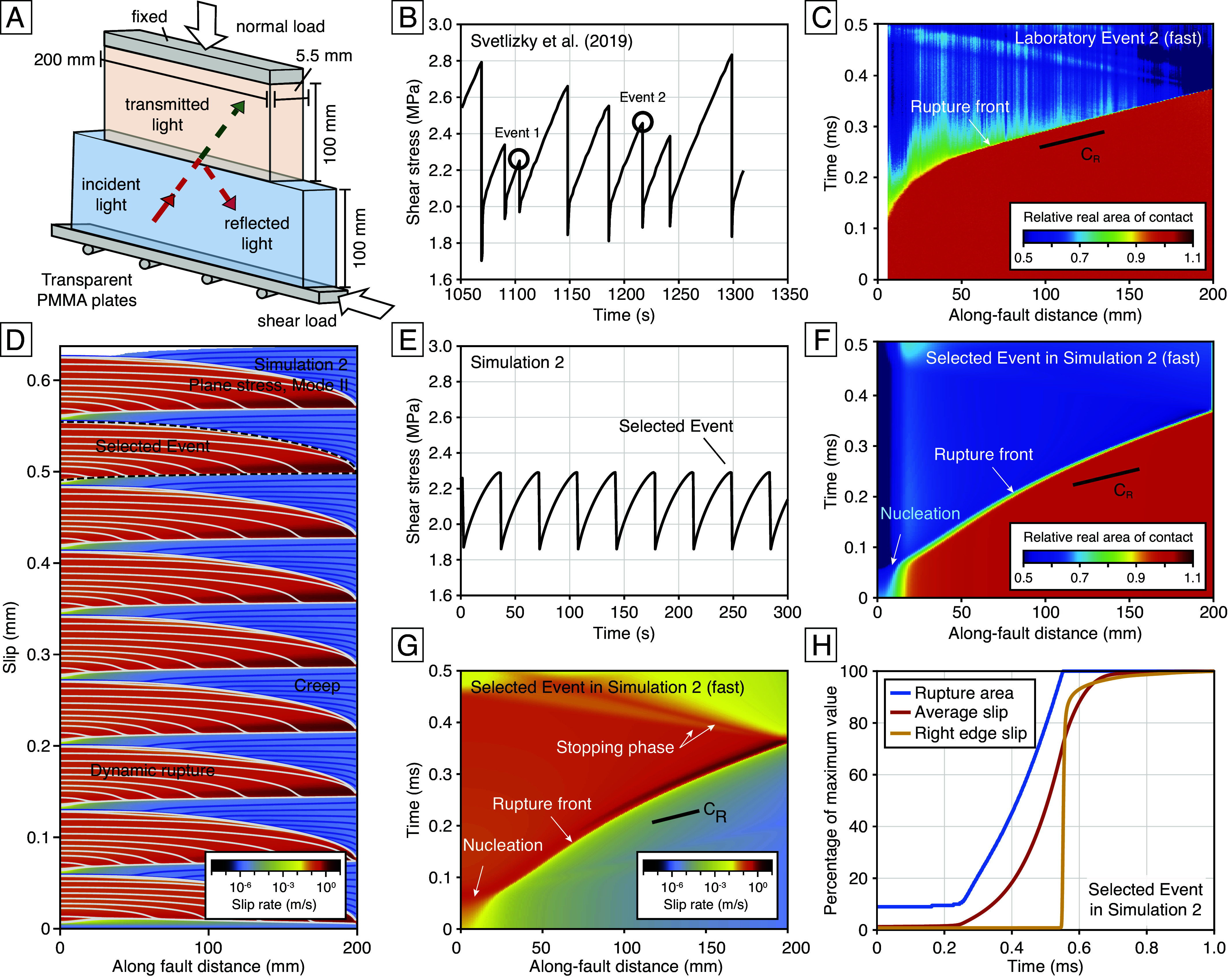
(*A*) Simplified schematic of the dynamic rupture experiment apparatus used to image the real area of contact (17). (*B*) ∼300-s average shear stress time series during the dynamic rupture experiments (17). (*C*) Spatiotemporal evolution of the real area of contact in Laboratory Event 2 (17). The $A_{r}$ time series at each coordinate is normalized to its initial value before the slip instability. (*D*) Fully dynamic seismic cycle simulation encompassing nine events from Simulation 2, color-coded by slip rates. White slip contours indicate periods when the maximum slip rate exceeds 1 cm/s, with a contour interval of 50 µs. Blue slip contours indicate periods when the maximum slip rate is less than 1 cm/s, with a contour interval of 4 s. (*E*) 300-s average shear stress time series in Simulation 2. (*F*) Spatiotemporal evolution of the real area of contact for the selected event in Simulation 2, using the same normalization as in (*C*). (*G*) Same event as (*F*) but showing the slip-rate evolution. (*H*) Time series of rupture area (blue), average slip (red), and slip at the fault’s right edge (yellow). All series are normalized by their maximum values at the end of the event.

**Fig. 4.**
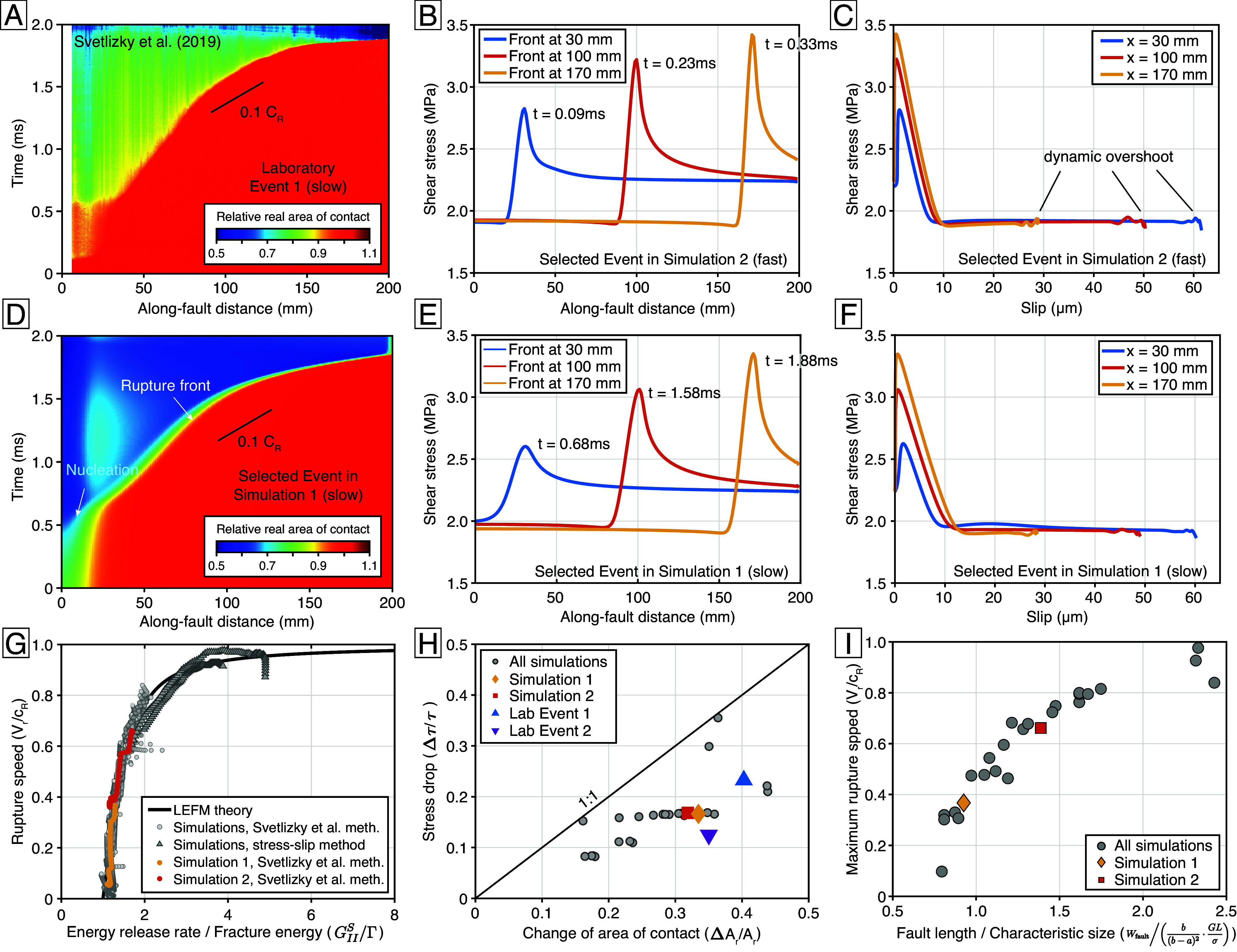
(*A*) Spatiotemporal evolution of the real area of contact in Laboratory Event 1 (slow). The time series of $A_{r}$ at each along-fault coordinate is normalized to its initial value before the onset of the slip instability. (*B*) Along-fault shear stress profiles during a single event for Simulation 2 (fast). Three profiles correspond to moments when the rupture front arrives at three specific locations. (*C*) Shear stress versus slip relation during a single event rupture for Simulation 2 (fast) at the same three locations as in (*B*). (*D*) Same as (*A*) but for a selected event in Simulation 1 (slow). (*E*) Along-fault shear stress profiles during a single event for Simulation 1 (slow). Three profiles correspond to moments when the rupture front arrives at three specific locations. (*F*) Shear stress versus slip relation during a single event rupture for Simulation 1 (slow) at the same three locations as in (*E*). (*G*) Analysis of the simulation results using linear elastic fracture mechanics (LEFM). The dots represent measurements from a single event in simulations (*SI Appendix*, Table S1). (*H*) Relative change in the average shear stress plotted against the relative change in the average real area of contact before and after a rupture. (*I*) Maximum rupture velocity during a single event in a simulation versus fault length W normalized to the intrinsic length scale $GbL/({\left(b-a\right)}^{2}\sigma )$ (56, 57).

We consider spontaneous dynamic rupture experiments on PMMA monitored by high-rate measurements of optical transmissivity, conducted by Svetlizky et al. (17). Two long and narrow acrylic plates are forced against each other with normal load from the *Top* (Fig. 3*A*). A shear force is applied from the side to induce sliding at the interface. As shear stress accumulates, a small perturbation normal to the sliding direction is applied on one end of the interface to initiate slip instability. By choosing the timing of perturbations, Svetlizky et al. (17) generate events with different average stress drops in the same sequence (Fig. 3*B*). Optical transmissivity is measured using a high-speed camera along the narrow rectangle interface and averaged along the width direction. Assuming a linear relationship between the real area of contact and the light intensity (25) and normalizing the light intensity by the value before each instability results in profiles of the relative real area of contact during dynamic ruptures. Figs. 3*C* and 4*A* show the contact area evolution of slow (Event 1) and fast (Event 2) ruptures within the sequence. Event 2 has a rupture speed close to the Rayleigh wave speed $c_{R}$. Event 1 has a rupture speed close to $0.1\;c_{R}$.

We model the laboratory observations using numerical simulations of dynamic ruptures that resolve all phases of the seismic cycle, including the radiation of seismic waves, based on the boundary element method (*SI Appendix*, *Text* 2). We simulate two-dimensional mode-iiruptures on a 200 mm fault assuming plane-stress conditions (*SI Appendix*, Fig. S1). The left end of the simulated fault is subject to a Neumann boundary condition, approximating a traction-free surface. For simplicity, we mainly explore the aging evolution law. The friction properties are set to be homogeneous, and a uniform long-term slip-rate is enforced along the fault. With this setup, episodic ruptures spontaneously nucleate, propagate, and stop. By trial and error, we found two aging-law parameter sets that can generate events similar to Events 1 and 2, which we refer to as Simulations 1 and 2. Ruptures propagate unilaterally from left to right due to the traction-free surface. All events in the same simulated sequence have identical rupture evolution (Fig. 3*D* and *SI Appendix*, Figs. S4*A* and S5) and average stress drop (Fig. 3*E* and *SI Appendix*, Fig. S4*B*). These parameters are constrained well considering the given laboratory conditions, initial and final stress of events, relative contact area drop, and rupture style. Some key parameters for both simulations are $\mu_{0}=0.45$, a=0.018, b=0.024, and σ=5 MPa. In Simulation 2, we have L=0.7µm, while in Simulation 1, L=1µm.

Fig. 3 *F* and *G* show the spatiotemporal evolution of the contact area and slip rate for a selected event in Simulation 2. The rapid contact area drop near the rupture front coincides with the rapid acceleration of slip. The rupture arrest on the side triggers backward propagating waves, similar to the laboratory observations. Svetlizky et al.’s (17) observations of macroscopic motion commencing only after these ruptures have traversed the entire interface can be explained in our simulation (Fig. 3*H*): The *Right* edge of the fault does not start slipping until the rupture front has passed by, and the average slip does not achieve its maximum value yet when the rupture has traversed the entire fault. Fig. 4*D* show the spatiotemporal evolution of contact area for a representative event in Simulation 1. With a slight increase in L from 0.7 to 1 µm, the rupture speed slows down from $0.6\;c_{R}$ to $0.1\;c_{R}$.

Our spontaneous rupture simulations simultaneously reproduce the gradual increase in rupture speed with distance as well as the approximately 30% drop in the real area of contact (Figs. 3*F* and 4*D*), as observed in the laboratory experiments (Figs. 3*C* and 4*A*). The aging-law simulations also reproduce the increase in relative real area of contact drop with distance, as shown in the real area of contact profiles at three different moments when the rupture front arrives at three locations (*SI Appendix*, Fig. S6). Considering the discrepancies in configuration between the laboratory and numerical experiments, our simulations explain the evolution of the real area of contact during laboratory ruptures reasonably well.

### Relationship with Linear Elastic Fracture Mechanics.

In the numerical simulations, the rupture speed in Simulations 1 and 2 differ because of the varying characteristic slip distance L. In contrast, Laboratory Events 1 and 2 have different rupture speeds and stress drops because of the timing of triggering. Yet, linear elastic fracture mechanics can explain both results. Fig. 4*B* shows three along-fault shear stress profiles in the single event in Simulation 2 (fast), corresponding to the moment when the rupture front arrives at three locations, propagating from *Left* to *Right*. The shear stress increases slowly ahead of the rupture front and drops abruptly behind to a residual baseline. In addition, the shear-stress versus slip relation at those three locations follows an abrupt weakening profile (Fig. 4*C*). These results suggest that the spontaneous events in the seismic cycle simulations can also be treated as mode-iifracture propagation, consistent with previous theoretical analyses (55, 56). The representative event in Simulation 1 (slow) has similar shear stress profiles and stress-slip relations, except with wider rupture front widths and a longer slip weakening distance (Fig. 4 *E* and *F*).

Svetlizky et al. (17) analyzed the laboratory ruptures with subshear and unilateral propagation using linear elastic fracture mechanics, which relates the local rupture speed $V_{r}$ to the ratio between local static energy release rate $G_{\mathrm{II}}^{S}$ and local fracture energy Γ. With strain measurements near the fault, they measure local stresses before and after rupture, with which $G_{\mathrm{II}}^{S}$ can be calculated. Assuming a linear slip weakening profile, local Γ can be estimated using the local stress and the $A_{r}$ profile. The local rupture speed predicted by $G_{\mathrm{II}}^{S}/\Gamma$ compares successfully against the rupture speed measurement made separately using the light intensity (17).

We perform the same fracture mechanics analysis on our synthetic ruptures (*SI Appendix*, *Text* 3). We analyzed 26 aging-law simulations that produce subshear unilateral ruptures in a single event (*SI Appendix*, Table S1). We use the same measurement methods as in ref. 17, except for two simulations with high rupture speed, where the stress-slip relations provide better estimates of the local fracture energy. The local rupture speeds in our synthetic events are well explained by linear elastic fracture mechanics (Fig. 4*G*). These results demonstrate that a point-wise constitutive friction law capable of generating rapid weakening, such as the one proposed here or others (27, 58–66), once integrated with a linear elastic medium and proper boundary conditions, can be consistent with linear elastic fracture mechanics descriptions of macroscopic behavior.

We conduct three additional simulations using the slip evolution law (*SI Appendix*, *Text* 5). The slip-law simulations exhibit slip-weakening behavior, similar to the aging-law simulations (*SI Appendix*, Figs. S7*G*, S8*G*, and S9*F*). However, the rupture transitions from slow nucleation to near-Rayleigh rupture speed over a very short distance, and the nucleation phase is bilateral and asymmetric (*SI Appendix*, Figs. S7*C*, S8*C*, and S9*C*). These characteristics of the slip-law have also been demonstrated in previous theoretical analyses (55, 57). The slip-law simulations cannot reproduce the unilateral rupture or the gradual increase in rupture speed with distance, both of which are key features observed in laboratory experiments (17). Therefore, we chose not to pursue a more in-depth analysis with the slip-law in this study. Nevertheless, slip-law simulations may reproduce laboratory results with more realistic model configurations.

Because the constitutive framework relates the macroscale rupture with micro-scale physical process, it provides additional explanatory power. For example, the relative drops of $A_{r}$ are greater than the relative drops of shear stress τ in the aging-law dynamic rupture experiments (Fig. 4*H*). As the fault is still slipping at a speed several orders greater than the initial speed after the drop of the area of contact (Fig. 3 *F* and *G*), the direct velocity-strengthening effect compensates a portion of the weakening caused by the area of contact drop. The compensating proportion may vary for different friction parameters, but it always makes the relative area of contact drop greater than the relative stress drop (Fig. 4*H*). Furthermore, the rupture styles are connected with the frictional parameters. The maximum rupture speed of the spontaneous ruptures in our aging-law simulations is controlled by the ratio between fault length W=200mm and a characteristic nucleation size derived from the quasi-static fracture mechanics analysis for two-dimensional rupture in an elastic full space, $GbL/({\left(b-a\right)}^{2}\sigma )$ (56, 57), where G is the shear modulus (Fig. 4*I*). This ratio reflects the elastic energy release rate relative to the fracture energy at the termination of the unilateral rupture. Consequently, a higher ratio corresponds to a greater maximum rupture speed.

## Discussion and Conclusion

We present a constitutive friction law that explains the coevolution of the effective friction coefficient and the real area of contact in a laboratory setting (17, 25). The model describes the transient evolution and steady-state value of the real area of contact in quasi-static experiments (25). Within seismic cycle simulations, the ruptures spontaneously follow stress-versus-slip profiles akin to linear slip weakening. Furthermore, the fracture energy and rupture speed follow the predictions of linear elastic fracture mechanics. The abrupt slip weakening profile is not prescribed in the constitutive law. Instead, it emerges when coupled with the elastic medium and the boundary conditions. After the termination of the macroscopic rupture, a residual strength subsists, compatible with a frictional behavior. In addition to explaining the source properties of slow and fast ruptures, the proposed model successfully predicts the amount of light transmitted across the frictional interface.

Fracture mechanics provides an integrated description of rupture characteristics based on the energy balance at the crack tip and a posteriori knowledge of the strength-versus-slip profile. In contrast, the constitutive framework provides a point-wise description of failure. In this context, the macroscopic behavior, characterized by rupture style, rupture propagation speed, and stress drop, emerges when coupled with elastodynamics. The macroscopic behavior depends additionally on the size of the fault, the distribution of prestress, and the nature of boundary conditions. There is no dichotomy between fracture mechanics and friction theories, although they might appear distinct (20, 67), and both frameworks provide useful descriptions of dynamic ruptures.

Early slip-rate- and state-dependent friction laws (5, 60, 68) were predominantly empirical, posing challenges for the extrapolation of constitutive behavior to conditions relevant to tectonic faulting. The constitutive framework and parameters presented in this study are constrained to isobaric, isothermal, and nominally dry conditions applicable to the limited range of slip rates and materials examined here. Under these conditions, the model aligns closely with earlier formulations (27, 58, 59). As the rheology of contact junctions is thermally activated (27, 58–66), our model can be extended to encompass a broader range of hydrothermal and barometric conditions to explain a wider range of experimental data (26, 45, 46, 69–72). Further investigation is needed to delineate the shared and distinct features among friction laws and to evaluate their efficacy across various scales and physical conditions.

Our findings demonstrate that the state variable is a measurable quantity, at least by proxy. The modulation of the real area of contact may affect other physical characteristics of laboratory faults, including porosity, hydraulic diffusivity, electrical conductivity, dilatancy, and the overall propagation of seismic waves (38–40, 42, 73, 74). In natural faults, additional processes may intervene, such as contact quality change (75), fault gouge deformation (66, 76–81), frictional melting (82), and formation of anisotropy in fault roughness (83). Active monitoring of faults based on such proxies may offer the opportunity to detect earthquake nucleation at an early stage, well before the radiation of seismic waves. Further laboratory and field studies should be conducted to test this hypothesis. With a deeper understanding of the underlying physics governing the evolution of fault friction and with adequate instrumentation, active monitoring of natural faults may provide useful tools to monitor and mitigate seismic hazards.

## Supplementary Material

Appendix 01 (PDF)

## Data Availability

Simulations, analysis, and scripts have been deposited in GitHub (https://github.com/boilingwu/ContactArea_PNAS). There are no data underlying this work. Previously published data were used for this work [Fig. 2 uses friction data published by Dieterich and Kilgore on Pure and Applied Geophysics, which we digitized from the original paper copy: (25). Figs. 3 and 4 use friction and contact area data published by Svetlizky et al. on Annual Review of Condensed Matter Physics, which the original authors shared. We have cited and acknowledged the paper and original authors accordingly: (17)].
