# Supplementary material for: Evolution of the real area of contact during laboratory earthquakes
Source: Proc Natl Acad Sci U S A. 2025 Jun 6;122(23):e2410496122. doi: 10.1073/pnas.2410496122 (PMC12167961; doi:10.1073/pnas.2410496122)
Supplement: Supplementary file 1 — Appendix 01 (PDF) [file pnas.2410496122.sapp.pdf]

Supporting Information for

Evolution of the Real Area of Contact during Laboratory Earthquakes

Baoning Wu, Sylvain Barbot

Baoning Wu, baoningw@usc.edu

This PDF file includes:

Supporting text
Figs. S1 to S9
Tables S1 to S2
SI References

Supporting Information Text

Spring-slider Model

We consider a quasi-static spring slider model to explain the laboratory data in (1). A slider is forced to slip by a loaded spring with a stiffness k. The free end of the spring moves at an imposed speed V_L. A quasi-static balance is required between the applied stress and the frictional resistance, leading to

$$\mu\sigma = -k\big(s - V_L t\big)\ , \tag{1}$$

where σ is the applied normal stress, assumed constant, μ is the coefficient of friction, and s represents the total slip of the slider. The effective friction coefficient μ is given by

$$\mu = \mu_0 \left(\frac{V}{V_0}\right)^{\frac{a}{\mu_0}} \left(\frac{\theta}{\theta_0}\right)^{\frac{b}{\mu_0}}\ , \tag{2}$$

where $V = \dot{s}$ is the slip rate of the slider, and θ is the state variable. To solve for the slip evolution, we take the time derivative of Eq. (2), to get

$$\frac{a}{\mu_0}\frac{\dot{V}}{V} + \frac{b}{\mu_0}\frac{\dot{\theta}}{\theta} = \frac{k}{\mu\sigma}\big(V_L - V\big)\ . \tag{3}$$

Combining Eq. (3) with a state evolution law (aging or slip law), we obtain a set of ordinary differential equations. We then solve for the slip and state evolution of the system using a fourth-order Runge-Kutta method, implemented in the `ode45` function in MATLAB R2023a.

Fully Dynamic Seismic-cycle Simulations

Simulations with the boundary element method. We use a fully dynamic boundary element method to simulate the laboratory dynamic ruptures. We consider a single fault plane embedded in an unbounded two-dimensional half-space governed by isotropic, uniform elasticity. We choose a coordinate system so that the x_1 direction aligns with the fault Γ (Figure S1). In this case, there is only one component of non-zero stress interaction, given by

$$\begin{aligned}\tau(\xi,t) &= \tau^0(\xi) - \frac{G}{2c_s}V(\xi,t)\\ &\quad + \int_\Gamma \mathrm{d}S(\xi')\int_0^t \mathrm{d}\tau \hat{K}(\xi,t-\tau;\xi',0)V(\xi',\tau) \quad (\xi,\xi'\in\Gamma),\end{aligned} \tag{4}$$

where $V(\xi,t)$ is the slip-rate, G here is the shear modulus, $-\frac{G}{2c_s}V(\xi,\tau)$ is the radiation damping term (2–4), and c_s is the shear wave speed. At the initial time $t=0$, no slip has occurred and the elastic stress on the fault is given by $\tau^0(\xi)$. $\hat{K}$ is the stress integral kernel. By equating Eq. (4) with the constitutive equation given in the main text, we may solve for the slip and stress evolution.

Our simulation encounters two possible states: quasi-dynamics and full dynamics. The stress interaction term in Eq. (4) can be separated into a static part and a dynamic part

$$\begin{aligned}\int_\Gamma \mathrm{d}S(\xi')\int_0^t \mathrm{d}\tau \hat{K}(\xi,t-\tau;\xi',0)V(\xi',\tau) &= \int_\Gamma \mathrm{d}S(\xi')\hat{K}_{\text{static}}(\xi;\xi')D(\xi')\\ &\quad + \int_\Gamma \mathrm{d}S(\xi')\int_0^t \mathrm{d}\tau \hat{K}_{\text{dynamic}}(\xi,t-\tau;\xi',0)V(\xi',\tau).\end{aligned} \tag{5}$$

When the dynamic part is not negligible, we consider the full stress interactions. Our numerical simulations use an explicit constant-time-step scheme during the dynamic state (5, 6). We adopt the discretization scheme in (7) and the corresponding discretized-form kernels for stress interactions.

During the inter-seismic period, the slip rate on the fault is low, and the stress contribution from the dynamic part is insignificant. Therefore, we adopt a quasi-dynamic approximation to save computational expense by dropping the dynamic part in our calculations (2). During the quasi-dynamic state, we use a fourth-order Runge-Kutta method to solve for the slip and stress evolution (8) with the `ode45` function in MATLAB R2023a. The transition between using the dynamic and quasi-dynamic solvers is controlled by a threshold of stress interaction difference between the fully-dynamic and quasi-dynamic solutions.

Our simulations use the following PMMA material parameters: shear wave speed $c_s = 1361$ m/s, longitudinal wave speed $c_l = 2680$ m/s, and density $\rho = 1170$ kg/m^3 (9, 10). We use the stress interaction kernels for the plane-strain condition (7). However, we scale the elastic properties to effectively simulate plate-stress conditions.

Boundary, discretization, and loading configurations. We simulate sequences of two-dimensional Mode-II ruptures along a 200 mm interface embedded in an elastic half-space (Figure S1). We approximate the half-space by incorporating a whole space along a 400 mm interface and enforcing a Neumann boundary condition at $x_1 = 0$ (Figure S1), with

$$V(x_1) = V(-x_1). \quad [6]$$

Due to the symmetry, the stress tensor component σ_{11} is zero at $x_1 = 0$; however, σ_{12} is not necessarily zero at $x_1 = 0$, and the boundary conditions are an approximation for free surface boundary conditions. We only interpret the simulation outputs on the fault at $x_1 > 0$.

The fault is discretized into 400 equal-size elements of 1 mm. All our simulations have a cohesive zone width and nucleation width of at least ~ 10 mm, which are well resolved by our spatial discretization. During the dynamic state, the time-step is fixed at $0.45 * (1 \text{ mm})/c_d^P$, corresponding to a Courant–Friedrichs–Lewy number of 0.45. This time-step size ensures that all dynamic waves are well resolved.

We apply a homogeneous and constant back-slip loading rate of V_L on the 400 mm fault, representing the quasi-static loading in the experiments (9). The shear loading rate is not given in (9). After some trial-and-error, we chose $V_L = 2\,\mu\text{m/s}$ so that the recurrence intervals are consistent with the laboratory experiments to the first order. $V_L = 2\,\mu\text{m/s}$ is also consistent with the typical loading in this type of experiments (10). The applied normal stress of 5 MPa is homogeneous and constant in all our simulations, the same as the reported value in (9).

Linear Elastic Fracture Mechanics Analysis in Our Simulations

In the main text, we apply the linear elastic fracture mechanics (LEFM) analysis from Svetlizky et al. (9) to our simulations. In the following supplementary text, we provide a summary of the analysis method used in Svetlizky et al. (9) (hereafter, referred to as Svet17) and describe the procedures employed to measure the relevant quantities in our simulations. The reference *Dynamic Fracture Mechanics* by Freund (11) will be frequently cited as Freund98. We note that Svet17's LEFM analysis assumes a time-independent loading behind the crack tip. In principle, approaches that are more suitable for the target crack-expansion configuration can be used by considering time-dependent loading behind the crack tip (12, 13). Nevertheless, we choose to adopt the time-independent approach since our main goal is to compare with Svet17's analysis.

Linear Elastic Fracture Mechanics Analysis in Svet17. In the experiments described in Svet17, ruptures propagate consistently from one side ($x = 0$ mm) to the other ($x = 200$ mm) (Figure 3a, 3c, 4a). Thus, this scenario can be treated as a Mode II crack growth problem, where a crack propagates from $x = 0$ mm to $x = 200$ mm along a prescribed fault. At a given crack length l, the rupture propagation speed is denoted by v (V_r in the main text).

According to linear elastic fracture mechanics theory, at the rupture front, the energy released due to stress reduction should balance the energy dissipated through the formation of new crack surfaces. This relationship is expressed

$$G = \Gamma \ , \quad [7]$$

where G represents the energy release rate at the rupture front, a function of the stress conditions and rupture speed. The energy dissipation rate at the rupture front Γ depends on the slip-weakening behavior at the rupture front.

The methodology in Svet17 involves several key steps: initially, the stress conditions and fracture energy at various locations within a given experiment are measured using strain gauge data and the cohesive zone width at the rupture front, determined from the real area of contact A_r profile. Subsequently, the spatio-temporal evolution of A_r is utilized to measure the local rupture propagation speed. The measurements of stress and fracture energy are independent of the local rupture propagation speed. LEFM predicts a relationship between these two independent sets of measurements. Svet17 demonstrates that the theoretical predictions of rupture speed, based on given stress and fracture energy, are consistent with their experimental data.

How does LEFM predict local rupture speed. To predict rupture propagation speed v with stress and fracture energy, we start with the energy release rate G at the rupture front. For a unilateral Mode II rupture, G is a function of rupture length l and rupture speed v, following

$$G = \frac{1-\nu^2}{E} A_{II}(v) K_{II}^2(l,v) \ , \quad [8]$$

where ν is the Poisson's ratio and E is Young's modulus. Eq. (8) is referred to as the 'Mode II' component of (5.3.10) in Freund98. $A_{II}(v)$ is a universal function that depends only on v, and its expression is given by (5.3.10) in Freund98. $K_{II}(l,v)$ is the Mode II stress intensity factor at the rupture front. Eq. (8) corresponds to Equation (1) in the supplementary material of Svet17. The differences are that Svet17 uses a plane-stress expression so there is no $1-\nu^2$ in the expression; also, they use the notation f_{II} instead of A_{II} for the universal function.

The stress intensity factor $K_{II}(l,v)$ can be decomposed as a rupture-speed-dependent term multiplying a static stress intensity factor,

$$K_{II}(l,v) = k_{II}(v) K_{II}^S(l) \quad [9]$$

where $k_{II}(v)$ is another universal function that depends only on v. $K_{II}^S(l)$ is a static stress intensity factor that depends on the crack length and the stress condition within the crack. Eq. (9) corresponds to Equation (3) in the supplementary material of

Svet17 and Equation (6.4.41) in Freund98. There are differences in equation appearance due to different choices of expressing arguments. Also, we follow Freund98 in using $k_{II}(v)$ to denote the universal function, while Svet17 uses κ_{II}.

If we denote $\Gamma(l)$ as the energy dissipation rate at crack length l (fracture energy), we may now combine Eqs. (7-9) to obtain

$$\begin{aligned}\Gamma(l) &= G(l,v) \\ &= \frac{1-\nu^2}{E}A_{II}(v)k_{II}^2(v)(K_{II}^S(l))^2 \\ &= g_{II}(v)G_{II}^S(l)\ ,\end{aligned} \quad [10]$$

where

$$\begin{aligned}g_{II}(v) &= A_{II}(v)k_{II}^2(v) \\ G_{II}^S(l) &= \frac{1-\nu^2}{E}(K_{II}^S(l))^2\ .\end{aligned} \quad [11]$$

Reorganizing Eq. (10), we derive the equation that predicts the rupture speed at the rupture front,

$$g_{II}(v) = \left(\frac{G_{II}^S(l)}{\Gamma(l)}\right)^{-1} \quad [12]$$

If we can measure the static energy release rate $G_{II}^S(l)$ and the fracture energy $\Gamma(l)$, we can use Eq. (12) to predict the local rupture speed v. The rupture speed v can also be independently measured using the A_r profiles. Svet17 found that the LEFM predictions are consistent with the independent v measurements.

Static energy release rate $G_{II}^S(l)$. To calculate $K_{II}^S(l)$ in Eq. (11), we use the expression given by Equation (4) in the supplementary material of Svet17,

$$K_{II}^S(l) = \frac{2}{\sqrt{\pi l}}\int_0^l \frac{\Delta\sigma_{xy}(s)F(s/l)}{\sqrt{1-(s/l)^2}}\,\mathrm{d}s\ , \quad [13]$$

where

$$F(s/l) = 1 + 0.3(1-(s/l)^{5/4})\ , \quad [14]$$

and $\Delta\sigma_{xy}(l)$ represents the initial shear stress minus the final shear stress at different fault locations. Eq. (14) was originally obtained from the edge crack stress intensity factor solution in *The Stress Analysis of Cracks Handbook* by Tada et al. (14), on page 197.

Fracture energy $\Gamma(l)$ using the Svet17 method. To calculate the fracture energy $\Gamma(l)$, Svet17 makes use of the equilibrium between the energy release rate G and fracture energy Γ,

$$\Gamma(l) = G(l) = \frac{1-\nu^2}{E}A_{II}(v)K_{II}^2(l,v)\ , \quad [15]$$

noting that we changed $1/E$ to $(1-\nu^2)/E$ compared to the expression just above Equation (6) in the supplementary materials of Svet17, for consistency in plane-strain notation.

Svet17 determines K_{II} using the stress and A_r information near the rupture front. LEFM suggests that the incremental stress field $\Delta\sigma_{ij}$ near the crack tip can be expressed as

$$\Delta\sigma_{ij} = \frac{K_{II}}{\sqrt{2\pi r}}\Sigma_{ij}^{II}(\theta, v) \quad [16]$$

where r and θ are the distance and angle relative to the crack tip. This equation is from Equation (1) in (15). Eq. (16) implies that the stress intensity factor K_{II} can be constrained if we measure how the stress decays with distance near the rupture front. Svet17 first measures the cohesive zone length (denoted as x_c) and the peak to residue shear stress (denoted as $\tau_p - \tau_r$). x_c can be measured with the A_r profile, assuming the cohesive zone is where A_r quickly drops. Once τ_p and x_c are measured at all the possible locations on the fault, they use Equation (6) in the supplementary materials to calculate the stress intensity factor K_{II},

$$K_{II} = (\tau_p - \tau_r)\sqrt{x_c}\sqrt{\frac{2}{\pi}}\int_{-\infty}^0 \frac{\widetilde{\tau}(\xi)}{-\xi}\,\mathrm{d}\xi, \quad [17]$$

where they use $\widetilde{\tau}(\xi) = e^{\xi}$. In this case, the integral can be analytically carried out and the answer is $\sqrt{\pi}$. Once they obtain the stress intensity factor K_{II}, they can use Eq. (15) to calculate $\Gamma(l)$.

As pointed out in Svet17, this method to measure $\Gamma(l)$ does not work when the local rupture speed is close to the Rayleigh wave speed c_R. Therefore, Svet17 only calculates $\Gamma(l)$ using experiments with low rupture speeds. They then apply the low-rupture-speed $\Gamma(l)$ to analyze the high-rupture-speed experiments, assuming that different experiments have the same $\Gamma(l)$ profile. This assumption is reasonable for their analysis because they used the same apparatus for all experiments. However, this is not a good assumption for our study, because simulations with different friction parameters exhibit different fracture energy profiles. In our study, we only use the Svet17 method to measure $\Gamma(l)$ for simulations with the maximum rupture speed being smaller than $0.8\,c_R$.

Fracture energy $\Gamma(l)$ estimate based on a stress-slip relation. For high-rupture-speed simulations, we may estimate the fracture energy using the stress-slip output at different locations. We use the following method to measure the fracture energy $\Gamma(l)$ from our simulations,

$$\Gamma(l) = \left(\int_0^{D_f(l)} \tau(l, s)\, \mathrm{d}s \right) - \tau_r(l) D_f(l), \tag{18}$$

where $D_f(l)$ is the slip when the fault shear stress at l finishes dropping and reaches a steady level (Figure 4c and 4f in the main text). In our analysis, we choose $D_f(l)$ as the final slip at fault location l.

Universal functions $A_{II}(v)$ and $k_{II}(v)$. To apply the theoretical relations to our simulations, we need to evaluate the so-called 'universal functions' $A_{II}(v)$ and $k_{II}(v)$. These two functions are referred to as f_{II} and κ_{II} in the supplementary material of Svet17. Svet17 did not provide details on how these two functions are calculated; instead, they refer readers to Freund98, which is a textbook. In the following section, we document how we use the formulae in Freund98 to calculate $A_{II}(v)$ and $k_{II}(v)$. The expression of $A_{II}(v)$ is given by Equation (5.3.11) in Freund98

$$A_{II}(v) = \frac{v^2 \alpha_s}{(1-\nu) c_s^2 D} \ , \tag{19}$$

where the expression for D can be found in the text between Equations (5.3.8) and (5.3.9),

$$D = 4\alpha_d \alpha_s - (1 + \alpha_s^2)^2 \ , \tag{20}$$

where ν is Poisson's ratio; c_d is the elastic dilatational wave speed (P wave speed), c_s is the elastic shear wave speed (S wave speed), and α_d and α_s are the quantities $\sqrt{1 - v^2/c_d^2}$ and $\sqrt{1 - v^2/c_s^2}$. Note that $A_{II}(v) \to 0$ when $v \to 0$. Since $D \to 0$ when $v \to c_R$, $A_{II}(v) \to \infty$ when $v \to c_R$. The expression of $k_{II}(v)$ is given by Equation (6.4.42) in Freund98,

$$k_{II}(v) = \frac{1 - c/h}{S_+(h)\sqrt{1 - b/h}} = \frac{1 - v/c_R}{S_+(v^{-1})\sqrt{1 - v/c_s}} \ , \tag{21}$$

where $h = 1/v$, as mentioned in the first paragraph on page 349 in Freund98, $a = 1/c_d$ (used to calculate $S_+(h)$), $b = 1/c_s$, and $c = 1/c_R$ are mentioned in the List of Symbols at the beginning of the book.

The function $S_+(h)$ is given by (6.4.18) in Freund98,

$$S_\pm(\zeta) = \exp\left\{ -\frac{1}{\pi} \int_{a_\mp}^{b_\mp} \arctan[V(\eta)]\, \frac{\mathrm{d}\eta}{\eta \pm \zeta} \right\} \ , \qquad V(\eta) = \left[\frac{4\eta^2 |\beta(\eta)| |\alpha(\eta)|}{(2\eta^2 - b^2 - b^2\eta^2/h^2 \mp 2b^2\eta/h)^2} \right] . \tag{22}$$

We note that the original expression of $V(\eta)$ in Freund98 was slightly different,

$$V(\eta) = \left[\frac{4\eta^2 \beta(\eta) |\alpha(\eta)|}{(2\eta^2 - b^2 - b^2\eta^2/h^2 \mp 2b^2\eta/h)^2} \right] ,$$

where the full complex number $\beta(\eta)$ was used, instead of only the amplitude. This may be a typographic error.

The definition of $a_\pm$ is given in the text below Equation (6.4.8),

$$a_\pm = a/(1 \pm a/h) \ . \tag{23}$$

Although we can't find the explicit definition of $b_\pm$, it can be inferred from the text nearby that $b_\pm$ should have a similar structure to $a_\pm$,

$$b_\pm = b/(1 \pm b/h). \tag{24}$$

The $\beta(\eta)$ and $\alpha(\eta)$ functions used in $V(\eta)$ are given by Equation (6.4.10) and the associated text, except that the arguments are notated by ζ instead,

$$\alpha(\zeta) = \left(a^2 - \zeta^2 + a^2\zeta^2/h^2 - 2a^2\zeta/h\right)^{1/2}, \qquad \beta(\zeta) = \left(b^2 - \zeta^2 + b^2\zeta^2/h^2 - 2b^2\zeta/h\right)^{1/2}. \tag{25}$$

Measuring the required quantities from our simulations. To calculate $G_{II}^S(l)$ and $\Gamma(l)$ using the Svet17 method, we need to measure the initial, peak, and final shear stress at each fault location, which we refer to as τ_0, τ_p, and τ_r, respectively. In our simulations, τ_0 and τ_p are measured as the initial and peak values of the shear stress time series (Figures 4b and 4e), respectively. τ_r is measured as the shear stress value at the middle of the flat part in the slip-weakening stress-slip curve (Figures 4c and 4f), to avoid the influence of dynamic overshoots. When using the stress-slip method to measure $\Gamma(l)$, we use the entire stress-slip curve and Eq. (18).

We also need to measure the cohesive zone length x_c in order to use Eq. (17) to calculate $\Gamma(l)$. Once we obtain $G_{II}^S(l)$ and $\Gamma(l)$, we need to independently measure rupture speed v to test the LEFM prediction. x_c and v can both be measured from the A_r profiles. Here we demonstrate how we measure these variables using an automated approach, with the selected event in Simulation 2 as the example.

The left-most portion of the fault is the nucleation zone; these locations do not have a well-defined rupture front and cohesive zone (Figure S2a). The upper and lower bounds in time for measurable A_r profiles can be empirically determined through trial and error after initial inspection. The bounds are shown by the blue dashed lines. Figure S2b shows the A_r profile along the white dashed line in Figure S2a. Rupture front and cohesive zone can be clearly identified by the zone where the normalized A_r sharply drops from unity to a lower level. To automatically determine the start and end location of a rupture front, we take the second-order spatial derivatives of the A_r profile, shown as the orange line in Figure S2b. The minimum value of the second-order spatial derivatives profile marks the location where A_r starts to drop. We take this location as the 'rupture front start' location. The maximum value of the profile marks the location where A_r finishes dropping. We take this location as the 'rupture front end' location.

Figure S2c plots the 'rupture front start' and 'rupture front end' locations at different timesteps. The measurements demonstrate how the rupture propagates from left to right. We may now calculate the local rupture speed v at each location using the spatial derivative of rupture time. The raw results use the difference in rupture time of neighboring elements (blue stars in Figure S2d). They show some discontinuous spatial patterns, probably due to numerical discretization. A smoothed v is shown by the blue solid curve. The rupture speed increases as the crack length increases, as expected.

Orange stars in Figure S2d show the corresponding cohesive zone width at each location. They are measured by taking the difference between 'rupture front start' and 'rupture front end' locations. The pattern appears to be a staircase, probably due to the spatial discretization of our numerical model. The cohesive width x_c decreases with crack length, which is consistent with what is expected from the LEFM theory.

LEFM analysis on individual events. We demonstrate the application of LEFM analysis on individual events with three examples: the selected event in Simulation 1, the selected event in Simulation 2, and an event in the simulation with a rupture propagation speed close to the Rayleigh wave speed c_R (Figures S3a - S3c). We calculate $G_{II}^S(l)$ and $\Gamma(l)$ for each simulation (Figures S3d - S3f). $\Gamma(l)$ is determined using both the Svet17 method and the stress-slip method. The independently measured local rupture speeds v are then compared with the LEFM predictions (Figures S3g - S3i).

The Svet17 and stress-slip methods predict similar levels of $\Gamma(l)$ when the rupture speed is low (Figures S3d, S3e). The $G_{II}^S(l)/\Gamma(l)$ results are also comparable for both methods, with the Svet17 method yielding results closer to the LEFM predictions. However, when the rupture speed is high, the Svet17 method produces abnormally high $\Gamma(l)$ measurements (Figure S3f). This is because the Svet17 method requires the local rupture speed as an input and depends on the universal function $A_{II}(v)$ (Eq. (15)). $A_{II}(v)$ increases significantly when the local rupture speed approaches the Rayleigh wave speed (Eq. (19)), amplifying the uncertainties in measuring simulation quantities. In these high-rupture-speed cases, the stress-slip method provides $G_{II}^S(l)/\Gamma(l)$ results that align more closely with the LEFM predictions (Figure S3i). In this study, we employ the Svet17 method for simulations with a maximum rupture propagation speed less than $0.8c_R$, and utilize the stress-slip method for simulations with a maximum rupture propagation speed greater than $0.8c_R$.

Derivation of Equation [3]

The constitutive framework in this study attributes the empirical state-dependency to the fluctuations of the real area of contact A_r during sliding (16–20). Models of this kind envision that contact junctions are constantly being created and destroyed during sliding, and the contact population has a finite average age θ. The empirical state variable, by design, serves as a measure of the average contact population age at any given moment during sliding (21), and its evolution can be described by a state evolution law. Assuming this average age θ represents the timespan available for the contact area to grow, we may estimate the real area of contact A_r using θ with an equation describing contact area growth.

Under the laboratory conditions we examine in this study, a commonly accepted empirical relation is that the real area of contact A_r grows logarithmically with the average contact population age θ,

$$A_r \sim \log \theta \ , \qquad [26]$$

Different physical models for friction have different approaches to derive this logarithmic growth. In this study, we adapt the approach in Barbot (20) to derive this relation. The real area of contact A_r can be related to the size of micro-asperities by

$$A_r = \frac{N}{\chi_n}\left(\frac{d}{d_0}\right)^{\alpha} \ , \qquad [27]$$

where d and d_0 are the characteristic radius of curvature of contact junctions and a reference value, respectively; N is the normal component of traction, positive for compression; χ_n is the indentation hardness; α is a power-law exponent relating the characteristic surface roughness scale with the real area of contact, and is a number smaller than unity (22, 23). The flattening rate $\dot{d}/d$ is inversely proportional to d^p. The relationship between contact age and micro-asperity size can be obtained by integrating the flattening rate over time (20),

$$\theta \approx \frac{d^p}{G} \ , \qquad [28]$$

where G is a reference rate of growth and p is a power-law exponent (20). Combining Eq. 27 and Eq. 28, we obtain

$$A_r = \frac{N}{\chi_n}\left(\frac{\theta}{\theta_0}\right)^{\frac{\alpha}{p}} \ , \qquad [29]$$

which is Equation [3] in the main text, where $\theta_0 = d_0^p/G$ is a reference time of contact, and α/p is a positive number significantly smaller than unity. Therefore, Equation [3] in the main text is consistent with the empirical logarithmic contact area growth,

$$A_r \approx \frac{N}{\chi_n}\left[1 + \frac{\alpha}{p}\log\left(\frac{\theta}{\theta_0}\right)\right] \ . \qquad [30]$$

Another popular model explicitly considers plastic creep at contact junctions (16–19). Following this approach, we may obtain,

$$A_r \approx A_{r0}\left[1 + m\log\left(1 + \frac{\theta}{\tau}\right)\right] \ , \qquad [31]$$

where A_{r0}, m and τ are reference real area of contact, contact growth rate, and characteristic cutoff time, following the notations in Baumberger and Caroli (19). The characteristic time τ is often considered significantly smaller than most relevant θ values, and the above relation is thus consistent with the empirical logarithmic contact area growth.

This alternative model is different from the model used in the current study. There is an ongoing discussion about the difference, but it is beyond the scope of this study. This difference is important to distinguish when θ is very small. Since our study only concerns situations where θ is sufficiently large, our analyses are not affected by this discrepancy.

Evolution laws may be derived from a physical model without involving contact age (24–26). Examples include derivations from the characteristic surface roughness scale d (20, 27–30). Although it is possible to infer a relation between contact age θ and the roughness scale d (20), we do not rely on a specific derivation or physical model linking physical parameters to θ in this manuscript.

Additional Slip-law Simulations of Dynamic Rupture Cycles

We conducted three additional dynamic rupture cycle simulations with the Slip evolution law, referred to as Slip-law Simulations 1, 2, and 3. Their results are shown in Figures S7, S8, and S9, respectively. These simulations have the same model configuration as in the aging-law simulations, and their friction parameters are provided in Table S2. Slip-law Simulations 1 and 2 have parameters identical to those in Simulations 1 and 2 (aging law) shown in the main text. Slip-law Simulation 3 has the same parameters as Slip-law Simulations 1 and 2, except that its $L = 1.4\,\mu$m, which is a slightly larger value. As in the aging-law simulations, all events in the same simulated sequence exhibit identical rupture evolution, except for the first event, which is slightly different due to the initial conditions. For each simulation, we selected one complete event towards the end of the simulation as the representative event for detailed analysis.

The slip-law simulations share many characteristics with the aging-law simulations, such as stress drop, contact area drop, and return intervals. Notably, they also exhibit slip-weakening behavior, similar to the aging-law simulations (Figures S7g, S8g, S9f). However, the characteristic slip-weakening distance in the slip-law simulations is approximately 2-3 times smaller than in the corresponding aging-law simulations. Additionally, the weakening curve in the slip-law simulations is less linear compared to the aging-law simulations. These two slip-weakening characteristics of the slip law are consistent with previous theoretical results by Bizzarri and Cocco (31).

A major difference between the two evolution laws is that, in the slip-law simulations, the rupture transitions from slow nucleation to near-Rayleigh rupture speed over a very short distance. Additionally, slip-law ruptures are not strictly unilateral; the nucleation phase is bilateral in an asymmetric manner (Figures S7c, S8c, S9c; the asymmetry in nucleation is not immediately obvious in Figure S7c, but we confirmed it through closer inspection). The fast transition and asymmetric nucleation have also been demonstrated by the 2D quasi-static analysis of Ampuero and Rubin (32). In Slip-law Simulations 1 and 2, rupture propagation is relatively unilateral. Therefore, we performed fracture mechanics analysis on these two simulations (Figures S7e and S8e). The rupture speed near the nucleation is not well predicted by the fracture mechanics model for unilateral rupture, likely due to the asymmetric nucleation. However, the rupture speeds farther from the nucleation are well explained by fracture mechanics predictions using the Stress-slip methods. As expected, the Svet17 method does not work well since the rupture speeds are close to the Rayleigh wave speed.

The additional slip-law simulations support the conclusion that the friction and fracture mechanics frameworks are not contradictory. However, the slip-law simulations cannot reproduce the unilateral rupture or the gradual increase in rupture speed with distance, both of which are key features observed in laboratory experiments (33). Therefore, we choose not to pursue

an in-depth analysis with the slip-law in the main text. Nevertheless, it is worth noting that our simulation configuration is simplified compared to laboratory conditions. It is possible that slip-law simulations could reproduce laboratory results when more realistic configurations are implemented. This is beyond the scope of the current study and could be a subject for future research.

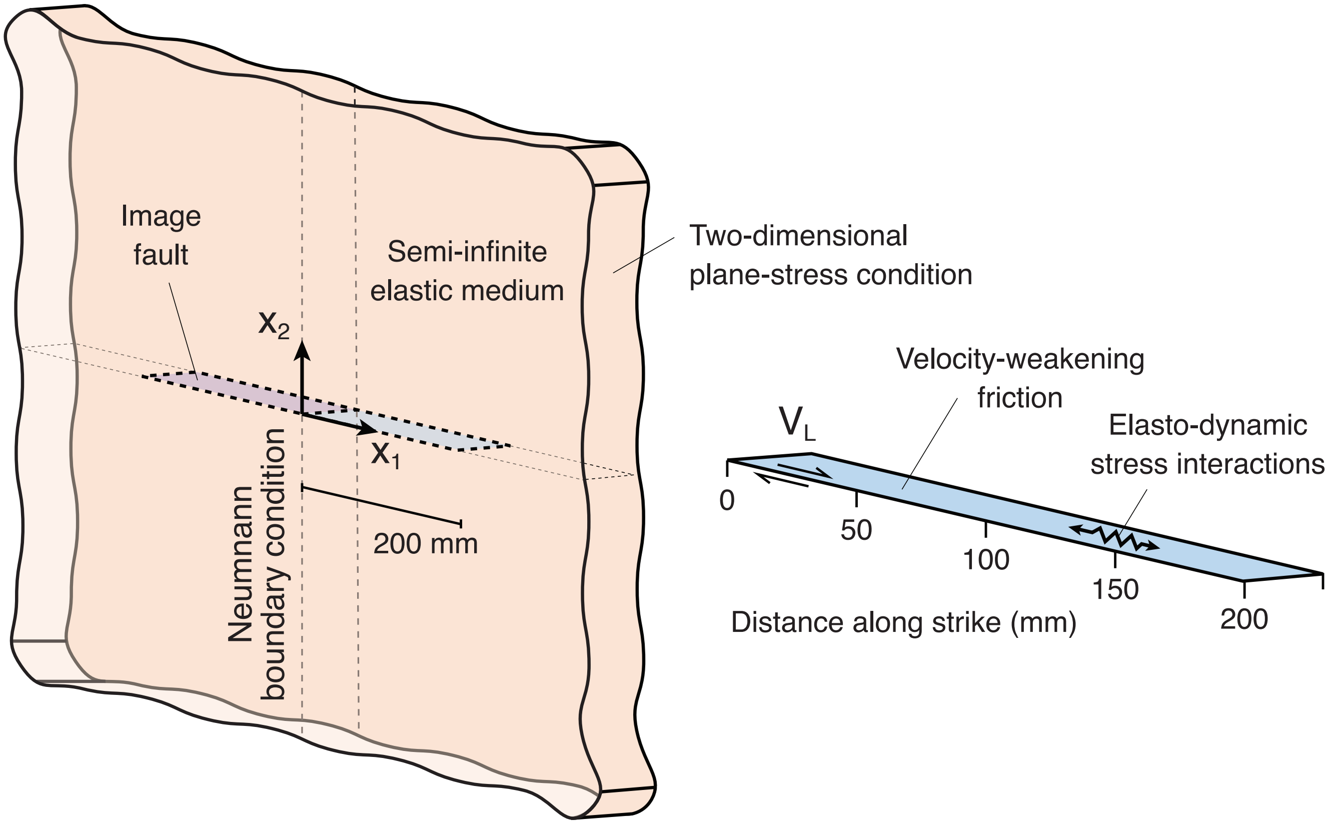

Fig. S1. Configuration of our dynamic rupture cycle simulations.

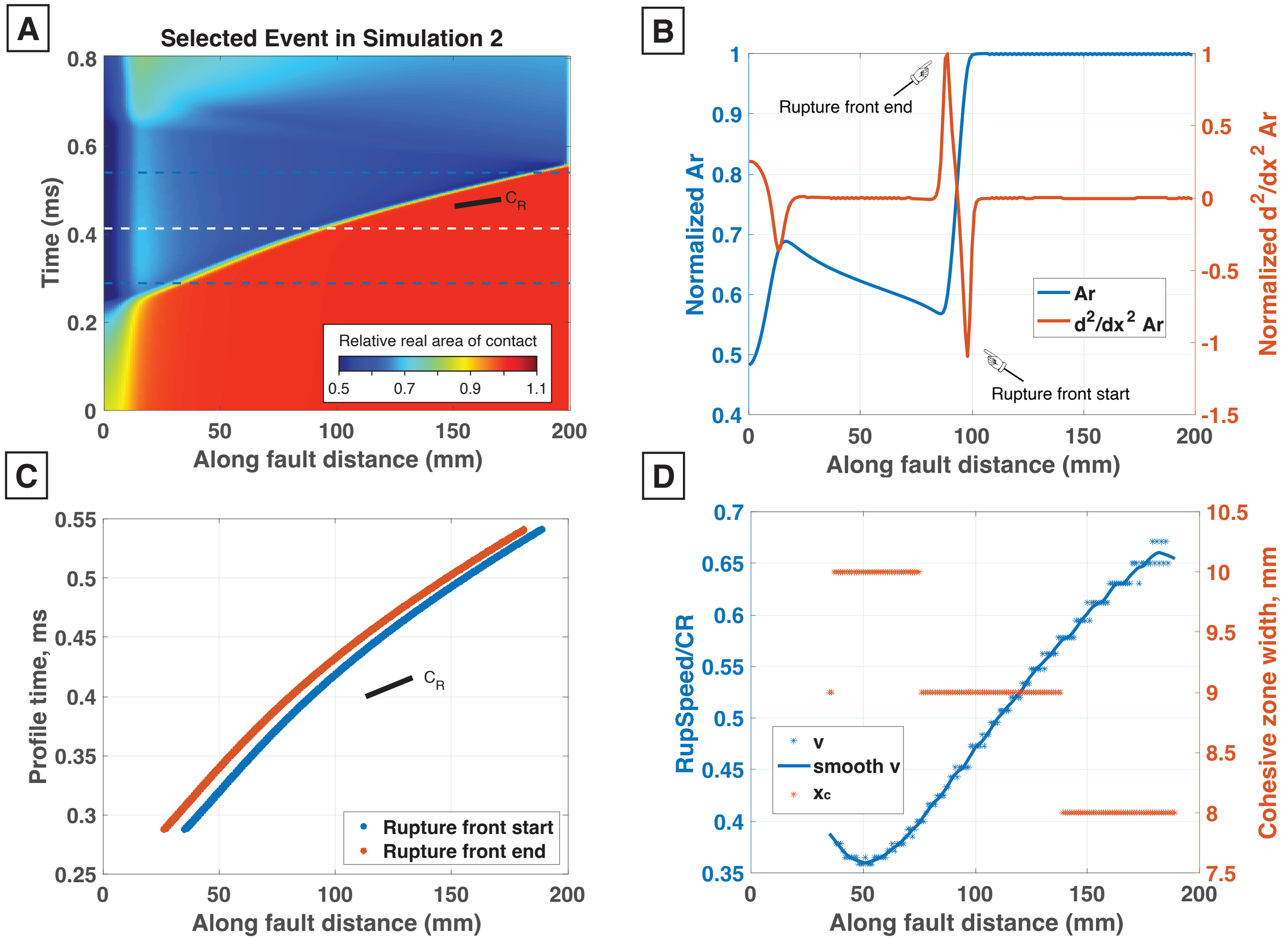

Fig. S2. **(a).** Spatio-temporal evolution of the real area of contact for the selected event in Simulation 2. The same as Figure 3f, but with a different y-axis range. Blue dashed lines show the time range where LEFM analysis is performed. The white dashed line shows the profile location of Figure S2b. **(b).** Normalized A_r profile (blue line) and its second-order spatial derivatives (orange line, normalized again) along the white dashed line in Figure S2a. **(c).** The 'rupture front start' (blue dots) and 'rupture front end' (orange dots) locations measured for each time step. **(d).** Rupture speed v and cohesive zone width x_c measured at different fault locations. Blue and orange stars are measurements of v and x_c at each location. Blue lines show the spatially smoothed v profile (smooth length 10 elements).

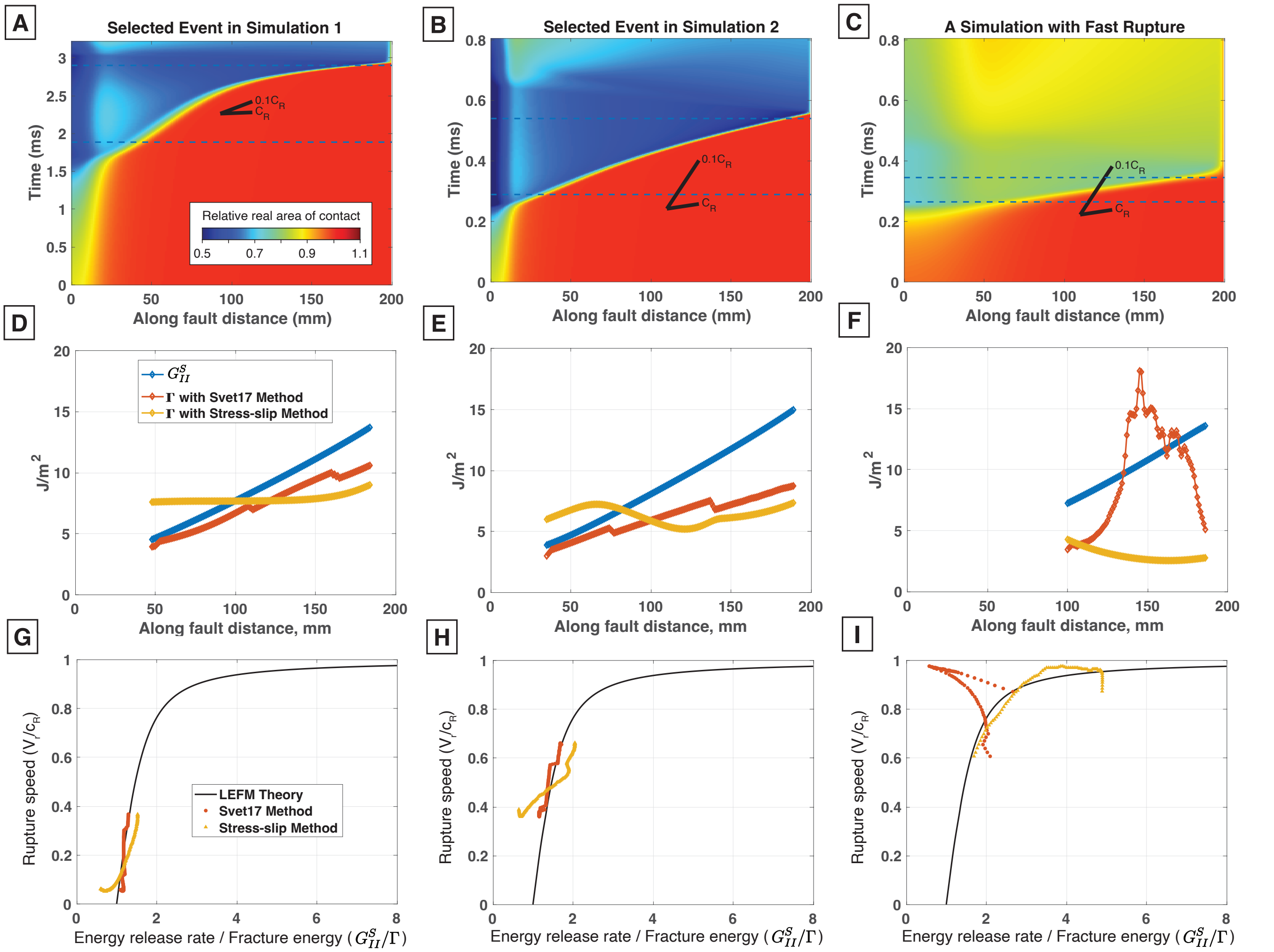

Fig. S3. (a)-(c). Spatio-temporal evolution of the real area of contact for individual events in three simulations. Blue dashed lines indicate the time range where LEFM analysis is performed. **(d)-(f).** Measurement of static energy release rate G^S_{II} and fracture energy Γ corresponding to the individual events in Figure S3a to S3c, respectively. G^S_{II} is depicted with blue curves. Γ measured with the Svet17 method is shown with red curves, while Γ measured with the stress-slip method is depicted with yellow curves. **(g)-(i).** Comparison between LEFM predictions of rupture speed v and the simulations, corresponding to the individual events in Figure S3a to S3c. Predictions with Γ measured by the Svet17 method are represented by red dots, and predictions with Γ measured by the Stress-slip method are denoted by yellow dots.

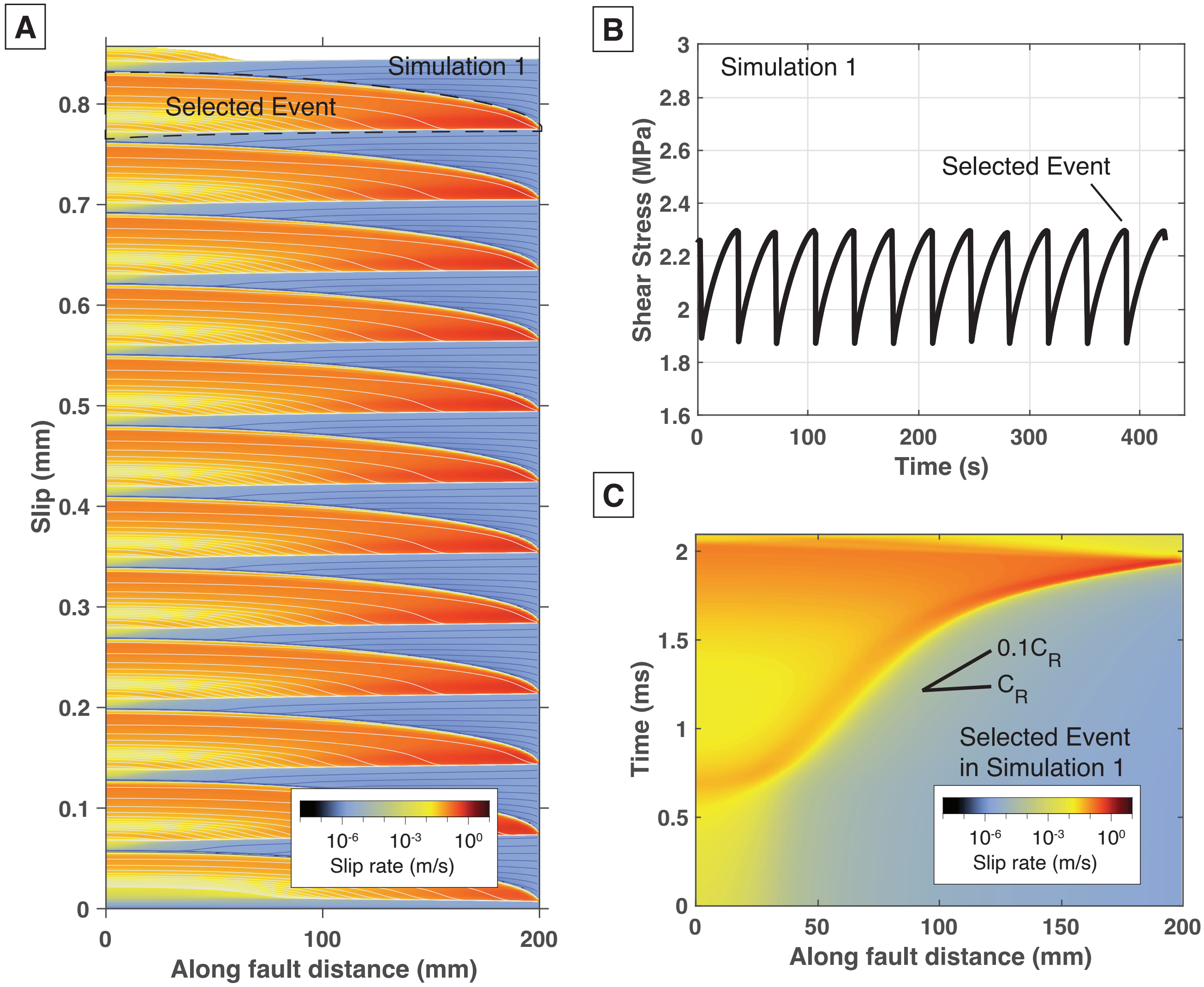

Fig. S4. (a). Fully dynamic seismic cycle simulation encompassing twelve events from Simulation 1, color-coded by slip rates. White slip contours indicate periods when the maximum slip rate exceeds 1 cm/s, with a contour interval of 100 µs. Noted that this is twice as much as the coseismic slip contour interval, 50 µs, in Figure 3d and Figure S5. Blue slip contours indicate periods when the maximum slip rate is less than 1 cm/s, with a contour interval of 4 s. **(b).** ∼420-second average shear stress time series in Simulation 1. **(c).** Same event as Figure 4d but showing the slip-rate evolution.

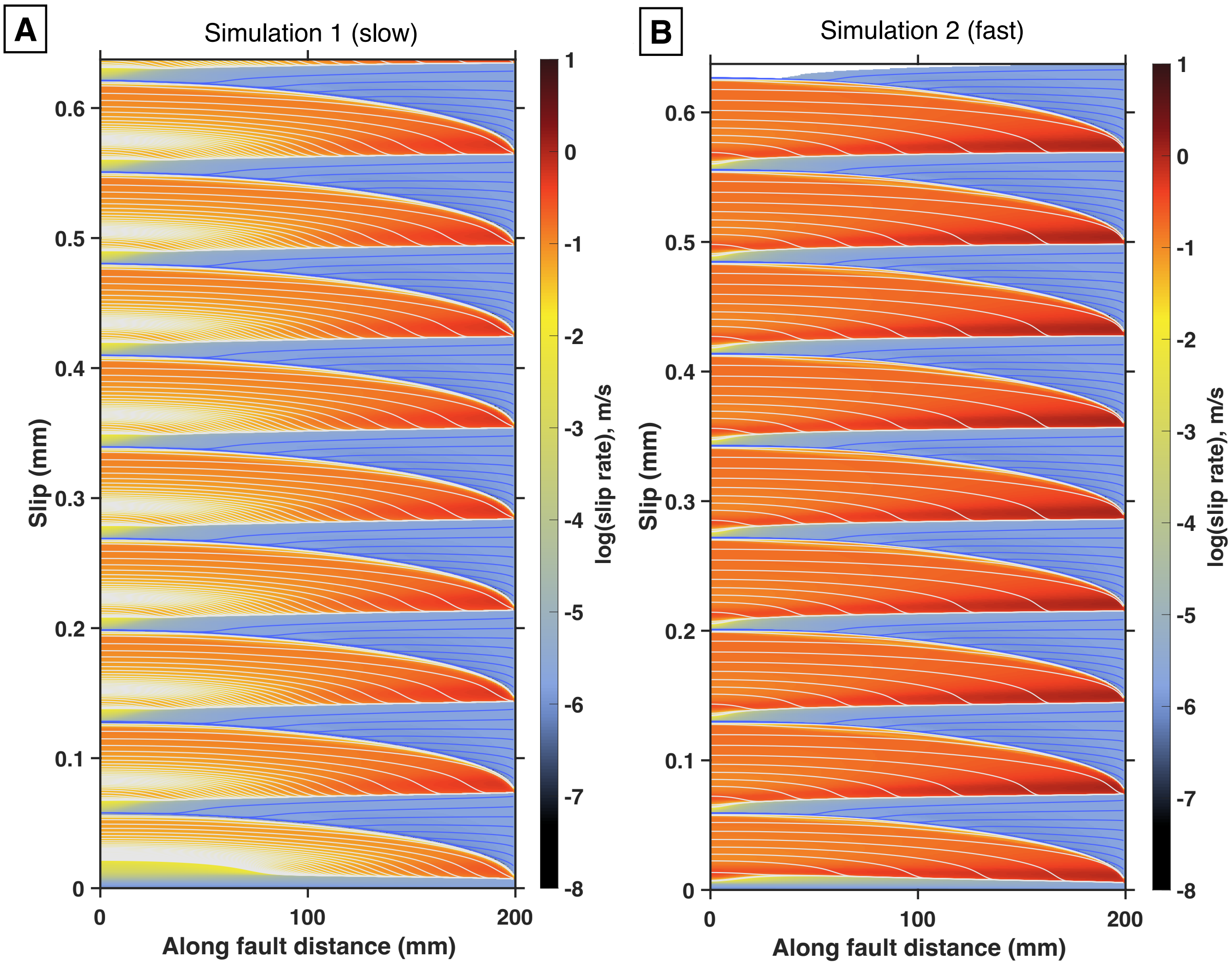

Fig. S5. Side-by-side comparison of slip contours between **(a)** Simulation 1 and **(b)** Simulation 2. White slip contours indicate periods when the maximum slip rate exceeds 1 cm/s, with a contour interval of 50 µs. Blue slip contours indicate periods when the maximum slip rate is less than 1 cm/s, with a contour interval of 4 s. The color represents slip rate. Only the initial 0.64 mm of slip are shown for both simulations.

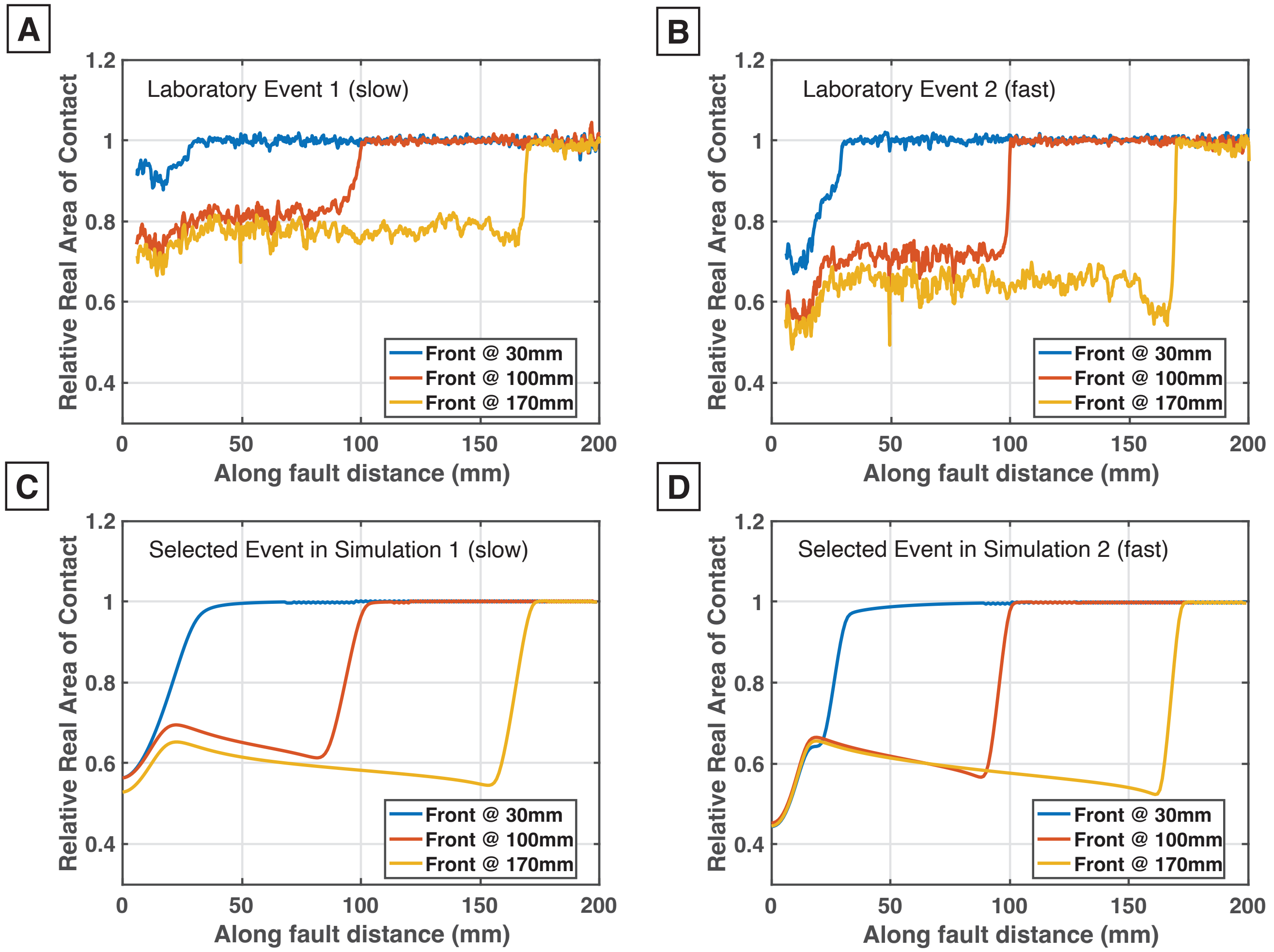

Fig. S6. Along-fault relative real area of contact profiles during a single event for **(a)** Laboratory Event 1, **(b)** Laboratory Event 2, **(c)** Selected Event in Simulation 1, and **(d)** Selected Event in Simulation 2, respectively. Three profiles correspond to moments when the rupture front arrives at three specific locations: $x = 30$ mm, $x = 100$ mm, and $x = 170$ mm.

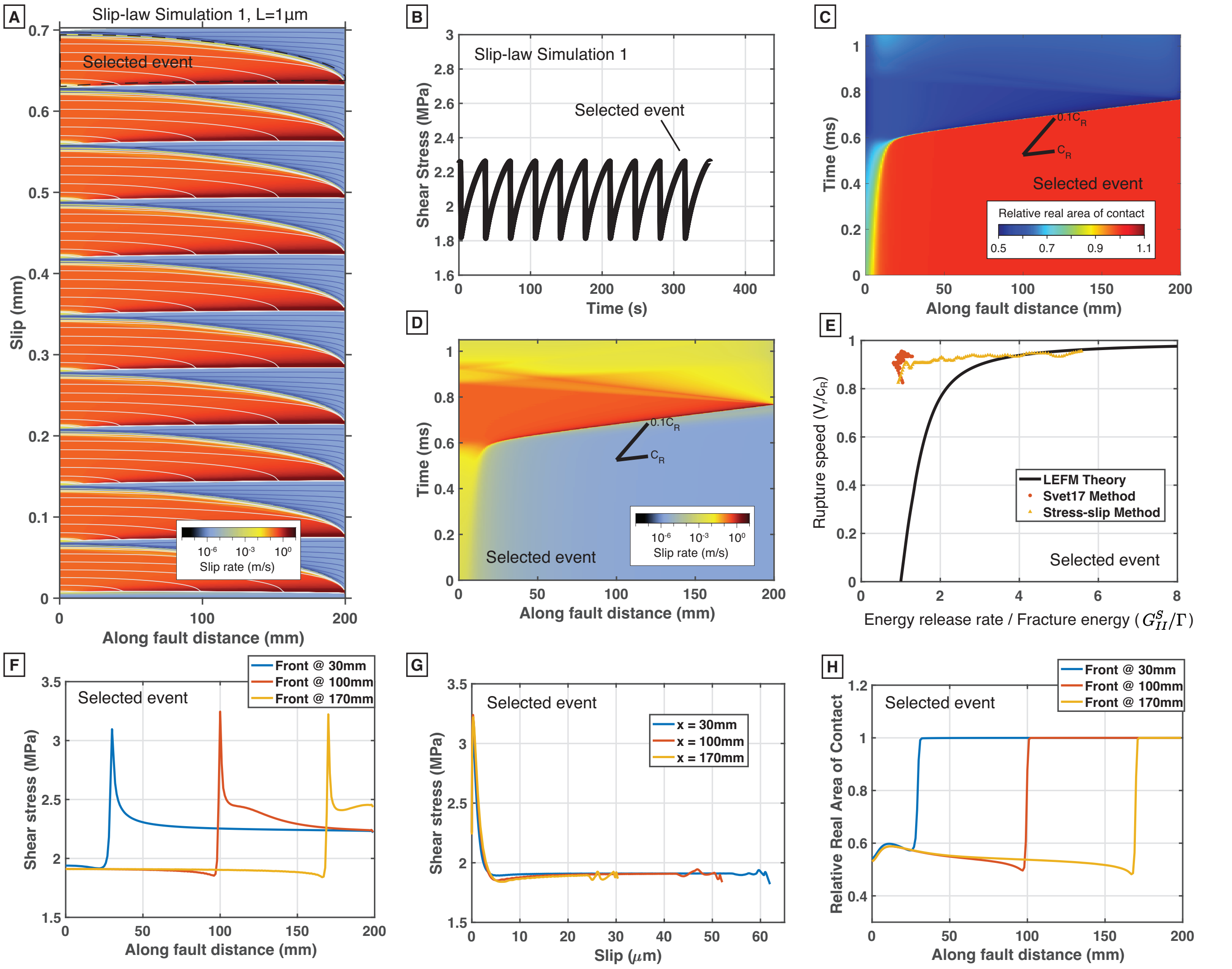

Fig. S7. Simulation results for the Simulation 1 parameters with the slip evolution law, referred to as Slip-law Simulation 1. **(a).** Slip contours of the simulation. White slip contours indicate periods when the maximum slip rate exceeds 1 cm/s, with a contour interval of 50 µs. Blue slip contours indicate periods when the maximum slip rate is less than 1 cm/s, with a contour interval of 4 s. The color represents slip rate. **(b).** Average shear stress time series in Slip-law Simulation 1. **(c).** Spatio-temporal evolution of the real area of contact for the selected event in Slip-law Simulation 1, using the same normalization as in Figure 3c and 3f. **(d).** Same event as (c) but showing the slip-rate evolution. **(e).** Comparison between LEFM predictions of rupture speed v and the selected event. Predictions with Γ measured by the Svet17 method are represented by red dots, and predictions with Γ measured by the Stress-slip method are denoted by yellow dots. **(f).** Along-fault shear stress profiles during the selected event. Three profiles correspond to moments when the rupture front arrives at three specific locations: $x = 30$ mm, $x = 100$ mm, and $x = 170$ mm. **(g).** Shear stress versus slip relation during the select event, at the same three locations as in (f). **(h).** Same as (f) but shows relative real area of contact profiles.

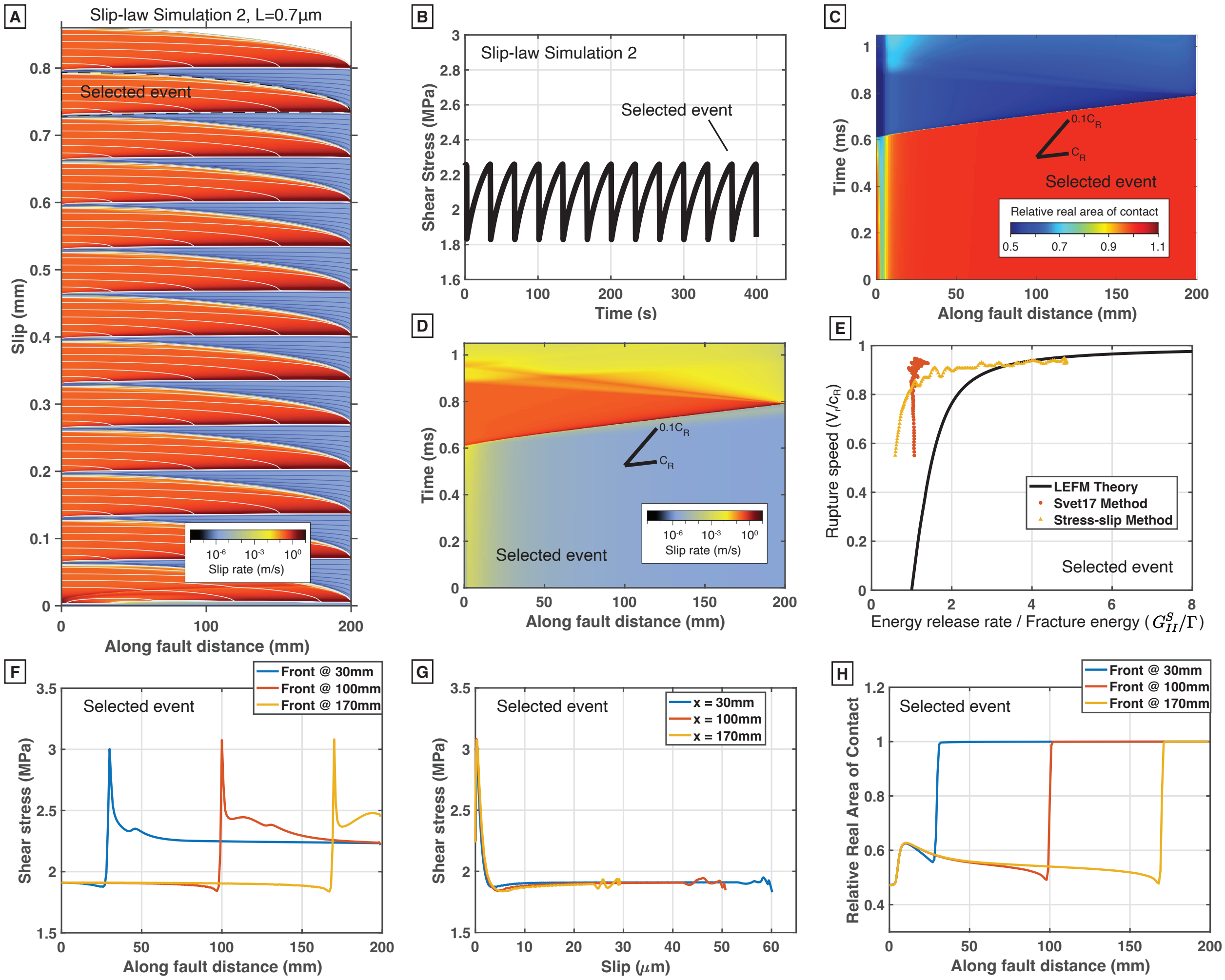

Fig. S8. Simulation results for the Simulation 2 parameters with the slip evolution law, referred to as Slip-law Simulation 2. **(a).** Slip contours of the simulation. White slip contours indicate periods when the maximum slip rate exceeds 1 cm/s, with a contour interval of 50 µs. Blue slip contours indicate periods when the maximum slip rate is less than 1 cm/s, with a contour interval of 4 s. The color represents slip rate. **(b).** Average shear stress time series in Slip-law Simulation 2. **(c).** Spatio-temporal evolution of the real area of contact for the selected event in Slip-law Simulation 2, using the same normalization as in Figure 3c and 3f. **(d).** Same event as (c) but showing the slip-rate evolution. **(e).** Comparison between LEFM predictions of rupture speed v and the selected event. Predictions with Γ measured by the Svet17 method are represented by red dots, and predictions with Γ measured by the Stress-slip method are denoted by yellow dots. **(f).** Along-fault shear stress profiles during the selected event. Three profiles correspond to moments when the rupture front arrives at three specific locations: $x = 30$ mm, $x = 100$ mm, and $x = 170$ mm. **(g).** Shear stress versus slip relation during the select event, at the same three locations as in (f). **(h).** Same as (f) but shows relative real area of contact profiles.

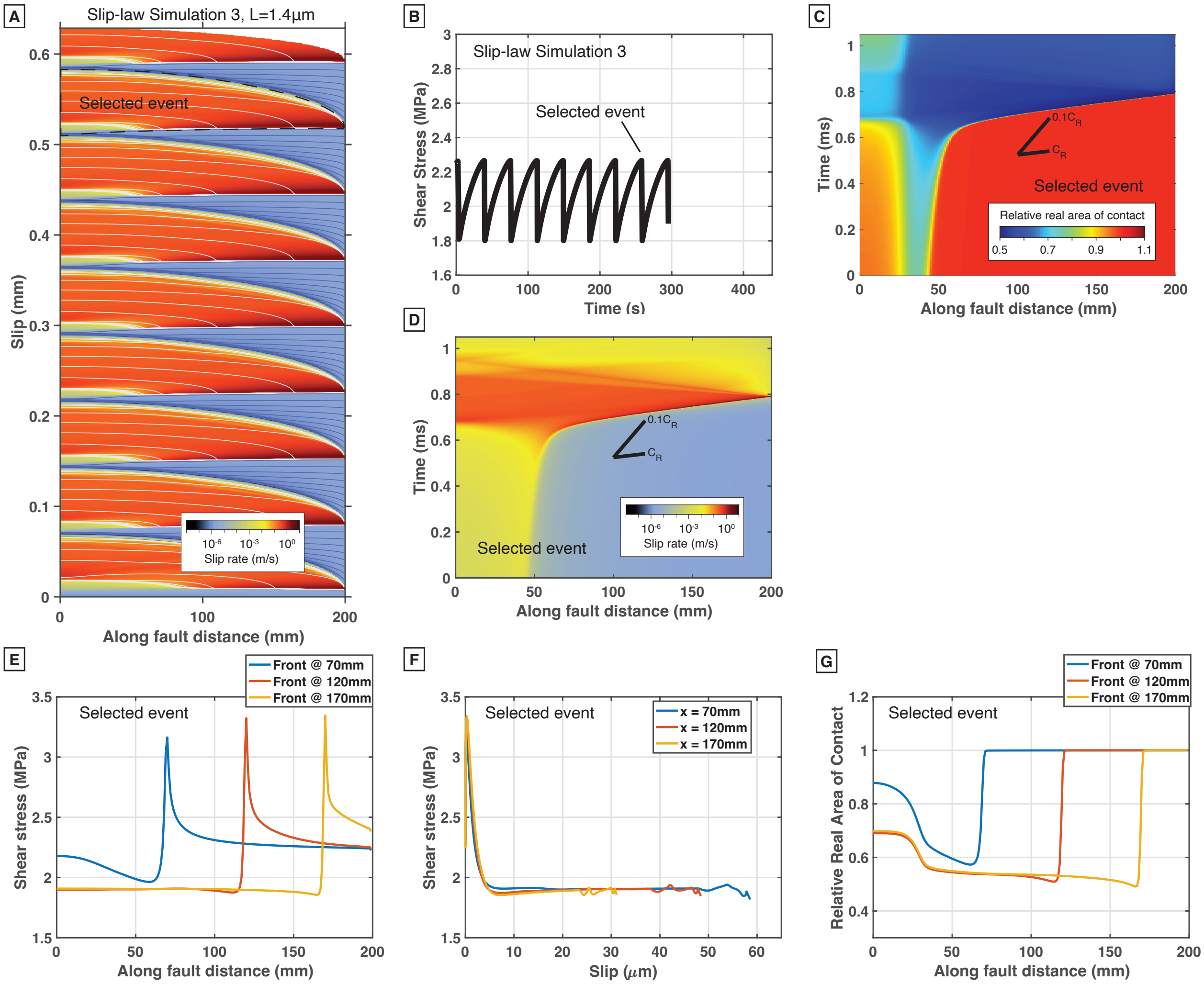

Fig. S9. Simulation results with the slip evolution law, where the parameters are the same as in Simulations 1 and 2, except that $L = 1.4, \mu\text{m}$, referred to as Slip-law Simulation 3. **(a).** Slip contours of the simulation. White slip contours indicate periods when the maximum slip rate exceeds 1 cm/s, with a contour interval of 50 µs. Blue slip contours indicate periods when the maximum slip rate is less than 1 cm/s, with a contour interval of 4 s. The color represents slip rate. **(b).** Average shear stress time series in Slip-law Simulation 3. **(c).** Spatio-temporal evolution of the real area of contact for the selected event in Slip-law Simulation 3, using the same normalization as in Figure 3c and 3f. **(d).** Same event as (c) but showing the slip-rate evolution. **(e).** Along-fault shear stress profiles during the selected event. Three profiles correspond to moments when the rupture front arrives at three specific locations: $x = 70$ mm, $x = 120$ mm, and $x = 170$ mm. **(f).** Shear stress versus slip relation during the select event, at the same three locations as in (e). **(g).** Same as (e) but shows relative real area of contact profiles.

Table S1. Fully-dynamic rupture cycle simulation parameters & results (aging law)

Note	a	b	L (μm)	$W_{\text{fault}} \Big/ \left(\frac{b}{(b-a)^2} \frac{GL}{\sigma} \right)$	Max v/c_R	$\Delta A_r/A_r$	$\Delta\tau/\tau$
Simulation 2	0.018	0.024	0.7	1.39	0.66	0.3186	0.1683
Simulation 1	0.018	0.024	1	0.93	0.37	0.3346	0.1662
	0.018	0.024	1	1.62	0.76	0.3085	0.1674
	0.009	0.012	0.5	0.97	0.47	0.1767	0.0837
	0.009	0.012	0.6	0.81	0.32	0.1794	0.0822
	0.009	0.012	0.4	1.21	0.68	0.1744	0.0837
	0.009	0.012	0.3	1.62	0.80	0.1643	0.0826
	0.012	0.016	0.8	0.81	0.30	0.2359	0.1096
	0.012	0.016	0.6	1.08	0.54	0.2318	0.1124
	0.012	0.016	0.4	1.62	0.80	0.2154	0.1114
	0.01	0.016	1	1.48	0.75	0.2453	0.1607
	0.008	0.014	1	1.67	0.80	0.2157	0.1586
	0.012	0.018	1	1.28	0.66	0.267	0.1636
	0.014	0.02	1	1.17	0.60	0.2907	0.165
	0.014	0.02	0.8	1.46	0.72	0.2815	0.1654
	0.016	0.022	1.2	0.87	0.33	0.3134	0.1629
	0.016	0.022	1	1.05	0.48	0.3143	0.1657
	0.016	0.022	0.8	1.31	0.68	0.3049	0.167
	0.016	0.022	0.6	1.75	0.82	0.2849	0.1653
	0.018	0.026	1	0.89	0.31	0.3586	0.1658
	0.018	0.026	0.8	1.12	0.49	0.3481	0.1687
	0.027	0.035	1.5	0.79	0.10	0.4386	0.2101
	0.027	0.035	1	1.19	0.46	0.4376	0.2214
	0.009	0.024	0.26	2.32	0.93	0.364	0.3552
Figure S3c	0.004	0.01	1	2.33	0.98	0.1614	0.1524
	0.012	0.024	1.6	2.43	0.84	0.3505	0.2989

Table S2. Additional Slip-law rupture cycle simulation parameters

Note	a	b	L (μm)
Slip-law Simulation 1	0.018	0.024	1
Slip-law Simulation 2	0.018	0.024	0.7
Slip-law Simulation 3	0.018	0.024	1.4

References

1. Dieterich JH, Kilgore BD (1994) Direct observation of frictional contacts: New insights for state-dependent properties. *pure and applied geophysics* 143(1):283–302.
2. Rice JR (1993) Spatio-temporal complexity of slip on a fault. *Journal of Geophysical Research: Solid Earth* 98(B6):9885–9907.
3. Cochard A, Madariaga R (1994) Dynamic faulting under rate-dependent friction. *pure and applied geophysics* 142(3):419–445.
4. Zheng G, Rice JR (1998) Conditions under which velocity-weakening friction allows a self-healing versus a cracklike mode of rupture. *Bulletin of the Seismological Society of America* 88(6):1466–1483.
5. Tada T (2009) Boundary integral equation method for earthquake rupture dynamics. *International Geophysics* 94:217–267.
6. Wu B (2021) Ph.D. thesis (University of California, Riverside).
7. Ando R, Kame N, Yamashita T (2007) An efficient boundary integral equation method applicable to the analysis of non-planar fault dynamics. *Earth, planets and space* 59:363–373.
8. Barbot S (2019) Slow-slip, slow earthquakes, period-two cycles, full and partial ruptures, and deterministic chaos in a single asperity fault. *Tectonophysics* 768:228171.
9. Svetlizky I, Kammer DS, Bayart E, Cohen G, Fineberg J (2017) Brittle fracture theory predicts the equation of motion of frictional rupture fronts. *Phys. Rev. Lett.* 118(12):125501.
10. Gvirtzman S, Fineberg J (2021) Nucleation fronts ignite the interface rupture that initiates frictional motion. *Nature Physics* 17(9):1037–1042.
11. Freund L (1998) *Dynamic Fracture Mechanics*, Cambridge Monographs on Mechanics. (Cambridge University Press).
12. Rice JR (1985) Three-dimensional elastic crack tip interactions with transformation strains and dislocations. *International Journal of Solids and Structures* 21(7):781–791. Topics in Continuum Mechanics.
13. Rice JR (1985) First-order variation in elastic fields due to variation in location of a planar crack front. *Journal of Applied Mechanics* 52(3):571–579.
14. Tada H, Paris PC, Irwin GR (2000) *The Stress Analysis of Cracks Handbook, Third Edition.* (ASME Press).
15. Svetlizky I, Fineberg J (2014) Classical shear cracks drive the onset of dry frictional motion. *Nature* 509(7499):205–208.
16. Brechet Y, Estrin Y (1994) The effect of strain rate sensitivity on dynamic friction of metals. *Scripta Metallurgica et Materialia;(United States)* 30(11).
17. Berthoud P, Baumberger T, G'Sell C, Hiver JM (1999) Physical analysis of the state- and rate-dependent friction law: Static friction. *Phys. Rev. B* 59(22):14313–14327.
18. Persson B (2000) *Sliding Friction: Physical Principles and Applications*, NanoScience and Technology. (Springer Berlin Heidelberg).
19. Baumberger T, Caroli C (2006) Solid friction from stick–slip down to pinning and aging. *Advances in Physics* 55(3-4):279–348.
20. Barbot S (2019) Modulation of fault strength during the seismic cycle by grain-size evolution around contact junctions. *Tectonophysics* 765:129–145.
21. Ruina A (1983) Slip instability and state variable friction laws. *Journal of Geophysical Research: Solid Earth* 88(B12):10359–10370.
22. Archard JF (1957) Elastic deformation and the laws of friction. *Proceedings of the Royal Society of London. Series A. Mathematical and Physical Sciences* 243(1233):190–205.
23. Greenwood JA, Williamson JBP (1966) Contact of nominally flat surfaces. *Proceedings of the Royal Society of London. Series A. Mathematical and Physical Sciences* 295(1442):300–319.
24. Sleep NH (2005) Physical basis of evolution laws for rate and state friction. *Geochemistry, Geophysics, Geosystems* 6(11).
25. Sleep NH (2006) Real contacts and evolution laws for rate and state friction. *Geochemistry, Geophysics, Geosystems* 7(8).
26. Chen J, Niemeijer AR (2017) Seismogenic potential of a gouge-filled fault and the criterion for its slip stability: Constraints from a microphysical model. *Journal of Geophysical Research: Solid Earth* 122(12):9658–9688.
27. Barbot S (2022) A rate-, state-, and temperature-dependent friction law with competing healing mechanisms. *Journal of Geophysical Research: Solid Earth* 127(11):e2022JB025106. e2022JB025106 2022JB025106.
28. Barbot S (2023) Constitutive behavior of rocks during the seismic cycle. *AGU Advances* 4(5):e2023AV000972. e2023AV000972 2023AV000972.
29. Barbot S (2024) Transient and steady-state friction in non-isobaric conditions. *Geochemistry, Geophysics, Geosystems* 25(2):e2023GC011279. e2023GC011279 2023GC011279.
30. Barbot S (2024) Does the direct effect of friction increase continuously with absolute temperature? *Proceedings of the National Academy of Sciences* 121(42):e2405111121.
31. Bizzarri A, Cocco M (2003) Slip-weakening behavior during the propagation of dynamic ruptures obeying rate- and state-dependent friction laws. *Journal of Geophysical Research: Solid Earth* 108(B8).
32. Ampuero JP, Rubin AM (2008) Earthquake nucleation on rate and state faults – aging and slip laws. *Journal of Geophysical Research: Solid Earth* 113(B1).
33. Svetlizky I, Bayart E, Fineberg J (2019) Brittle fracture theory describes the onset of frictional motion. *Annual Review of Condensed Matter Physics* 10(1):253–273.
